# Cyclic Behavior and Failure Modes of Q1100 Ultra-High-Strength Steel Box-Section Beam-Columns with Multiple Weld Repair Histories

**DOI:** 10.3390/ma19214733

**Published:** 2026-11-07

**Authors:** Chengzhong Gui, Lihao Chen, Yanmin Zhang, Yuxin Zhao, Quanhao Dong, Yuehang Zhang

**Affiliations:** 1 School of Earthquake Engineering and Building Safety, University of Emergency Management, Sanhe 065201, China; 2 College of Civil Engineering and Architecture, Zhejiang University, Hangzhou 310058, China; 3 Key Laboratory of Building Failure Mechanism and Disaster Prevention, China Earthquake Administration, Sanhe 065201, China; 4 Hebei Technology Innovation Centre for Multi-Hazard Resilience and Emergency Handling of Engineering Structures, Sanhe 065201, China; 5 School of Civil Engineering, Beijing Jiaotong University, Haidian District, Beijing 100044, China

**Keywords:** ultra-high strength steel, Q1100, box-section column, weld repair, hysteretic behavior, stability verification

## Abstract

Welding repair is often unavoidable when steel structures in seismic regions are damaged, yet how it affects the cyclic performance of ultra-high-strength steel (UHSS) members is still not well understood. In this study, the hysteretic behaviour and failure modes of Q1100 UHSS welded box-section beam-columns with extended flanges were examined experimentally, considering several weld-repair histories and strengthening measures. Ten test schemes from the SA, SB and SC series were subjected to a constant axial compression together with quasi-static cyclic lateral loading. The variables covered the number of repair passes (0–3), the repair technique, the axial load ratio (0.057–0.216), the steel grade (Q1100 versus Q345), the boundary restraint and the cross-sectional geometry. The Q1100 specimens failed in a fracture-dominated mode that began in the lower plate/weld region, whereas the Q345 specimen failed by buckling followed by tearing. In the SA and SB series, repeated repair tended to reduce the hysteretic energy dissipation and stiffness and to give asymmetric responses; these trends are consistent with cumulative welding damage, although the damage itself was not characterized. Appropriate strengthening combined with lateral restraint either restored or improved the deformation capacity, and the ultimate drift ratios lay between 2% and 9%. The skeleton curves ranged from an almost linear-elastic shape to an S-shape, depending on ductility, axial load and restraint. Extended flanges improved the lateral resistance, while unequal flange widths produced an asymmetric response. When checked against JGJ/T 483-2020, several Q1100 specimens satisfied the strength criterion but exceeded the in-plane or out-of-plane stability limits, which suggests that stability governed a number of the repaired schemes. No metallographic, hardness, residual-stress or fractographic analysis was carried out, so the damage mechanisms proposed here remain interpretive. Even so, these observations offer preliminary evidence for post-earthquake repair strategies and for design checks of Q1100 UHSS members of the type tested.

## 1. Introduction

The growing demand for lightweight, sustainable infrastructure has driven the adoption of ultra-high strength steels (UHSS) in civil engineering. Among them, Q1100 steel with a nominal yield strength of 1100 MPa offers substantial reductions in self-weight, material use, and carbon emissions, making it highly attractive for high-rise buildings and long-span bridges [1,2]. However, its application in seismic regions is constrained by pronounced cyclic softening and by a hysteretic response that is less stable than that of conventional structural steels [1,2]; this observation pertains to the deformation behavior reported in the material and member tests cited and to the tested members considered in this study, and should not be interpreted as evidence of intrinsic differences in material ductility. Box-section columns are widely employed in steel moment frames due to their high torsional stiffness and biaxial bending efficiency, and the incorporation of extended flanges can further enhance flexural capacity and joint performance.

At the material level, Zhang et al. [1] conducted cyclic coupon tests on Q1100 UHSS and reported rapid initial softening that stabilizes after a few cycles; the material was found to be nearly isotropic, and a combined hardening constitutive model was calibrated for numerical simulations. At the member level, cyclic tests on Q1100 H-section welded columns under combined compression and bending [2,3] identified the width-to-thickness ratio and axial compression ratio as the dominant parameters governing hysteretic behavior, and the applicability of Eurocode 3 (EN 1993-1-12) [4] slenderness limits was assessed. The flexural response of Q1100 welded I-sections was systematically investigated through coupon tests, residual stress measurements, and three-point bending experiments, leading to modified section classification limits and direct strength formulations [5]. For other high-strength and ultra-high-strength steels, further studies have clarified the cyclic characteristics. Experiments on Q690D beam-columns demonstrated significant cyclic softening and satisfactory energy dissipation, with ultimate inter-storey drift ratios (DRs) exceeding 1/50 [6,7], while studies on S690 steel [8] highlighted ductility retention under cyclic strains up to ±10% but an increased fracture risk at high strain amplitudes. Numerical studies incorporating cyclic softening and ductile fracture models have corroborated these findings [9,10], and Li et al. [11] observed negligible residual stress effects on the hysteretic behavior of Q460C steel. Wang et al. [12] emphasized that compact cross-sections are essential for maintaining moment capacity under large inelastic cyclic rotations. The strain-rate sensitivity and nonlinear stress–strain characteristics of UHSS necessitate tailored constitutive models for seismic analysis [10,13]. Despite these advances, current design codes, including Eurocode 3 (EN 1993-1-12) [4] and JGJ/T 483-2020 [14], were formulated—for steels with yield strengths not exceeding 700 MPa, and their validity for Q1100 UHSS members, especially after repair, remains uncertain.

Weld repair is an unavoidable operation during the service life of steel structures, but the additional thermal cycles introduced can irreversibly degrade the heat-affected zone (HAZ) microstructure. For ferritic steels, grain coarsening, hardness gradients, and residual stress accumulation are well-documented degradation mechanisms [15,16]. Lant et al. [17] reviewed practical weld repair procedures for low-alloy ferritic steels, including half-bead, temper-bead, and high-nickel filler techniques, aimed at minimizing subsequent cracking. Cold repair methods without post-weld heat treatment have been proposed as alternatives to reduce residual stresses and operational costs [18]. For Q1100 UHSS specifically, welding thermal simulation has been employed to construct continuous cooling transformation (SH-CCT) diagrams [19] and to systematically investigate the microstructural evolution and mechanical properties of the coarse-grained WAZ under various cooling rates [20]. In the context of fatigue, Al-Karawi [15] compiled approximately 130 test results on TIG-remelted welded details and demonstrated that even with a remaining crack depth of 2 mm, the fatigue life of repaired details could exceed the design life of new joints. A review of 311 published fatigue test results for TIG-dressed welds in steels with yield strengths of 272–1100 MPa further confirmed that TIG dressing markedly improves the fatigue strength of welded details, with the improvement becoming more pronounced as the steel yield strength increases [16]. Nevertheless, the influence of single and multiple repair passes on the subsequent cyclic response of Q1100 UHSS weldments has not been experimentally quantified.

Section slenderness and axial load level are key determinants of the cyclic behavior of HSS and UHSS beam-columns, primarily through their influence on local buckling and plastic deformation capacity. Higher width-to-thickness ratios accelerate local buckling and reduce deformation capacity in Q690D beam-columns [6,7] and in Q460 and Q690 box-section columns [21,22]. For UHSS grades such as S960 and Q1100, Su et al. [23] and Yin et al. [24] confirmed that codified slenderness limits for cross-section classification remain applicable, although compressive residual stresses can exacerbate buckling risks. Parametric studies have shown that axial load ratios exceeding 0.35 significantly degrade cyclic strength and energy dissipation in Q690D columns, particularly when combined with slender flanges or webs [9,25]. The role of extended flanges in mitigating local buckling has not been explicitly examined, yet related investigations offer indirect insights. Su et al. [26] proposed revised Eurocode interaction curves for S960 UHSS beam-columns, emphasizing enhanced flange stability to improve bending resistance. Li et al. [27] observed that welded H-sections with optimized flange dimensions exhibited lower compressive residual stresses, delaying buckling initiation. Studies on cold-formed G550 built-up sections [28] and square concrete-filled UHSS tubes at low temperatures [29] further indicated that flange geometry modifications can reduce local buckling susceptibility and weld fracture risk. Collectively, these findings suggest that extended flanges may serve as an effective strategy to improve the cyclic stability of UHSS members [23,27].

This study reports an experimental programme on ten test schemes carried out on four full-scale welded box-section beam-columns (three Q1100 and one Q345) with extended flanges, which had been subjected to different weld-repair histories and strengthening arrangements. The test matrix varied the number of repair passes (up to three), the repair and strengthening method (re-welding, stiffener addition, double bottom plates and inserted end welding), the axial load ratio (0.057–0.216), the steel grade (Q1100 versus Q345), the boundary condition (laterally free versus top lateral contact restraint) and the section geometry (including asymmetric extended flanges). The results are used to examine the failure modes, hysteretic response, energy dissipation and deformation limits, and the strength and stability provisions of JGJ/T 483-2020 [14] are then applied to the repaired Q1100 members, together with the resulting design implications. The aim is to provide a preliminary basis for the post-earthquake repair and seismic assessment of Q1100 UHSS structures, while recognizing the confounding among variables and the absence of microstructural evidence.

## 2. Experimental Program

### 2.1. Material Properties

The test specimens used two steel grades, Q1100 and Q345 (Table 1). For the Q1100 steel, the yield strength and ultimate tensile strength were provided by the manufacturer’s factory inspection report as 1210 MPa and 1470 MPa, respectively; the elastic modulus and Poisson’s ratio were approximately 206 GPa and 0.3, respectively. For the Q345 steel, the yield strength was determined according to GB 50017 [30] and JGJ/T483 [14] as 345 MPa, and this value was used in the axial load ratio *n* = *N*/(*Af_y_*) and in the code checks.

### 2.2. Specimen Design

**Figure 1:**
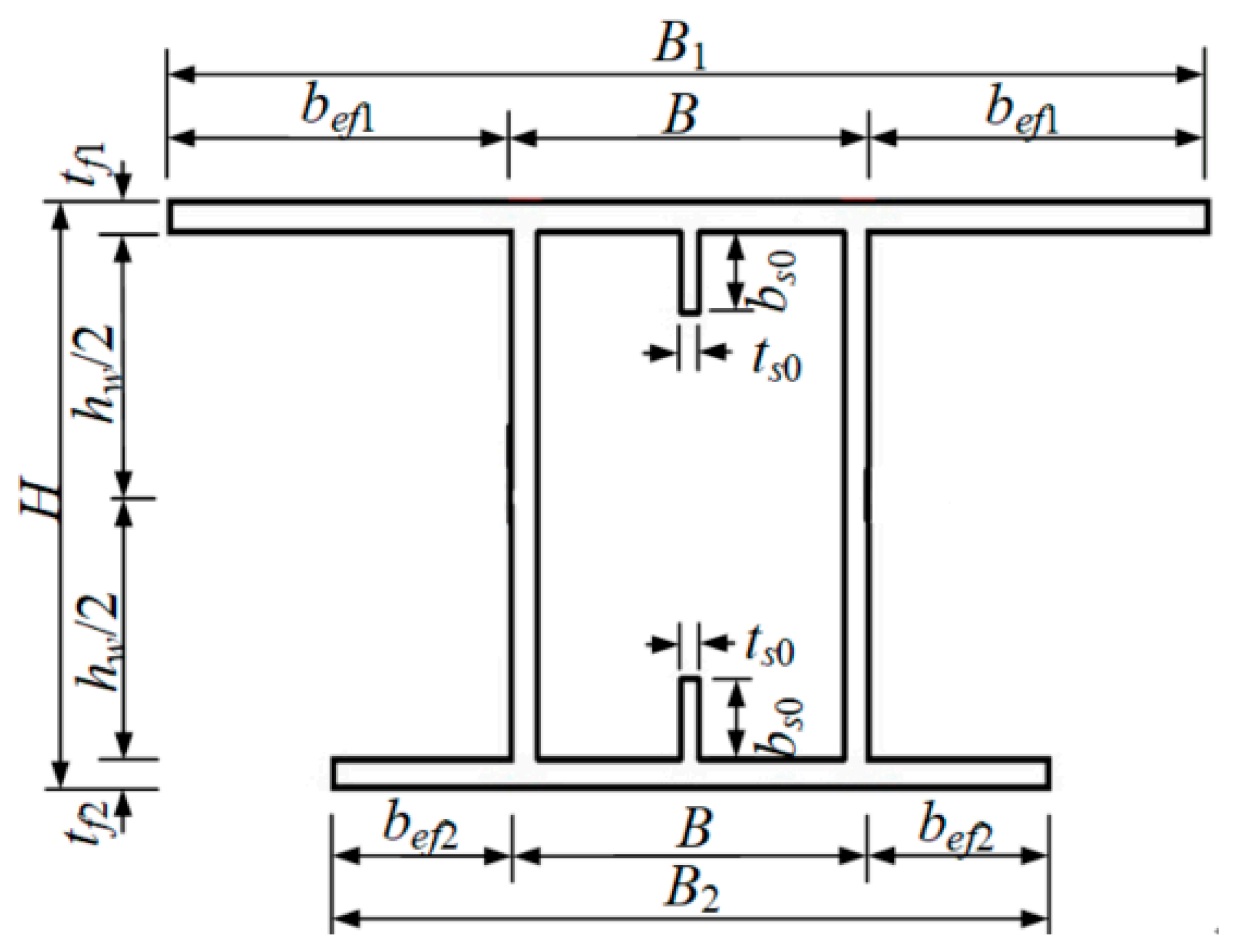
Diagram of the tested specimen dimensions.

The tests were performed on welded steel box compression–bending specimens with outwardly extended flanges, which were subjected to a small constant axial force and cyclic bending. Section dimensions in Figure 1 are defined by *H*, *B*, *t*, *b_ef_*_1_, *b_ef_*_2_, *b_s_*_0_, and *t_s_*_0_, representing section height, box width, flange thickness, upper and lower extended flange widths, stiffener plate width, and stiffener plate thickness, respectively; *t_f_*_1_ and *t_f_*_2_ are the upper and lower flange thicknesses. Measured dimensions are listed in Table 2. Ten test schemes were grouped into SA, SB, and SC series: SA has a 112 mm flange width, SB has equal 222 mm flange widths, and SC has unequal flange widths of 222 mm and 322 mm. The axial load ratio *n = N*/(*A·f_y_*) with the steel grades (Q345 vs. Q1100) ranges from 0.057 to 0.216, covering low-to-medium compression levels. The cross-sectional areas are 4360 mm^2^ for the SA series, 6120 mm^2^ for the SB series, and 6920 mm^2^ for the SC series.

**Figure 2:**
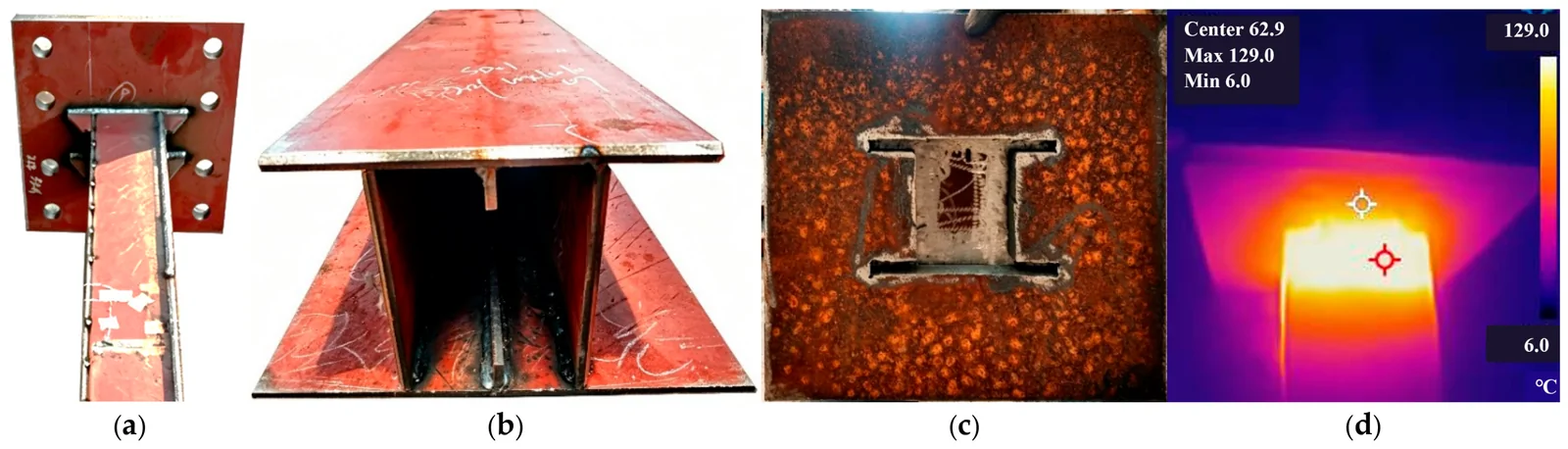
Cross-sectional welding details: (**a**) SA specimen; (**b**) SB specimen; (**c**) upper bottom plate; (**d**) weld-affected zone (thermal image; the colour scale denotes surface temperature).

Figure 2a,b presents the cross-sectional welding details of the SA and SB specimens. Before welding, 8 mm plates were first cut by CNC plasma cutting, and 45° bevels were prepared at both flange ends. CO_2_ gas-shielded flat welding was used. End plates of 25 mm and 35 mm thickness were welded to the upper and lower ends, respectively, and the longitudinal stiffeners at mid-width of the flanges were first tack-welded, then continuously welded along their full length. Temporary fixtures were used throughout to restrain welding distortion. The webs were then tack-welded longitudinally to the flanges and continuously welded. All welding used a special high-strength welding wire. For the “inserted end welding + DBS” scheme, the upper bottom plate had a cutout matching the external profile of the extended-flange box member, as shown in Figure 2c. Another 20 mm thick upper bottom plate was hollowed to fit the outer contour (Figure 2c). The member was first welded to the lower bottom plate and stiffeners; the upper bottom plate was then slipped over the member and fully welded to complete the assembly. Figure 2d shows the temperature field recorded while the end plate was welded to the longitudinal Q1100 and Q345 plates. Final fracture was consistently located in the lower plate/weld region. The thermal image documents the welding temperature field only, so WAZ embrittlement remains a possible but unverified explanation. The final fracture was consistently located at the lower plate/weld region. The thermal image in Figure 2d documents the welding temperature field only. WAZ embrittlement is a possible explanation but was not directly characterized.

Table 3 summarizes the complete specimen matrix and identifies the physical-member continuity. Ten test schemes were conducted on four physical members: SA-Q1100 (Nos. 1–4), SB-Q1100 (Nos. 5–8), SB-Q345 (No. 9), and SC-Q1100 (No. 10). In the SA series, Nos. 1–3 share the same axial load, but the same physical member is reused, and No. 4 simultaneously changes repair history, strengthening, and axial load. In the SB-Q1100 series, the axial load changes from 800 to 1450 kN after the first repair; Nos. 5–8 also differ in strengthening and boundary condition. The SB-Q345 and SC specimens differ from the SB-Q1100 specimens in grade, axial load ratio, geometry, and boundary condition. Consequently, the discussion below is framed in terms of associations within the tested matrix, not as controlled proof of the effect of repair count alone.

### 2.3. Test Setup and Instrumentation

#### 2.3.1. Loading System Configuration

**Figure 3:**
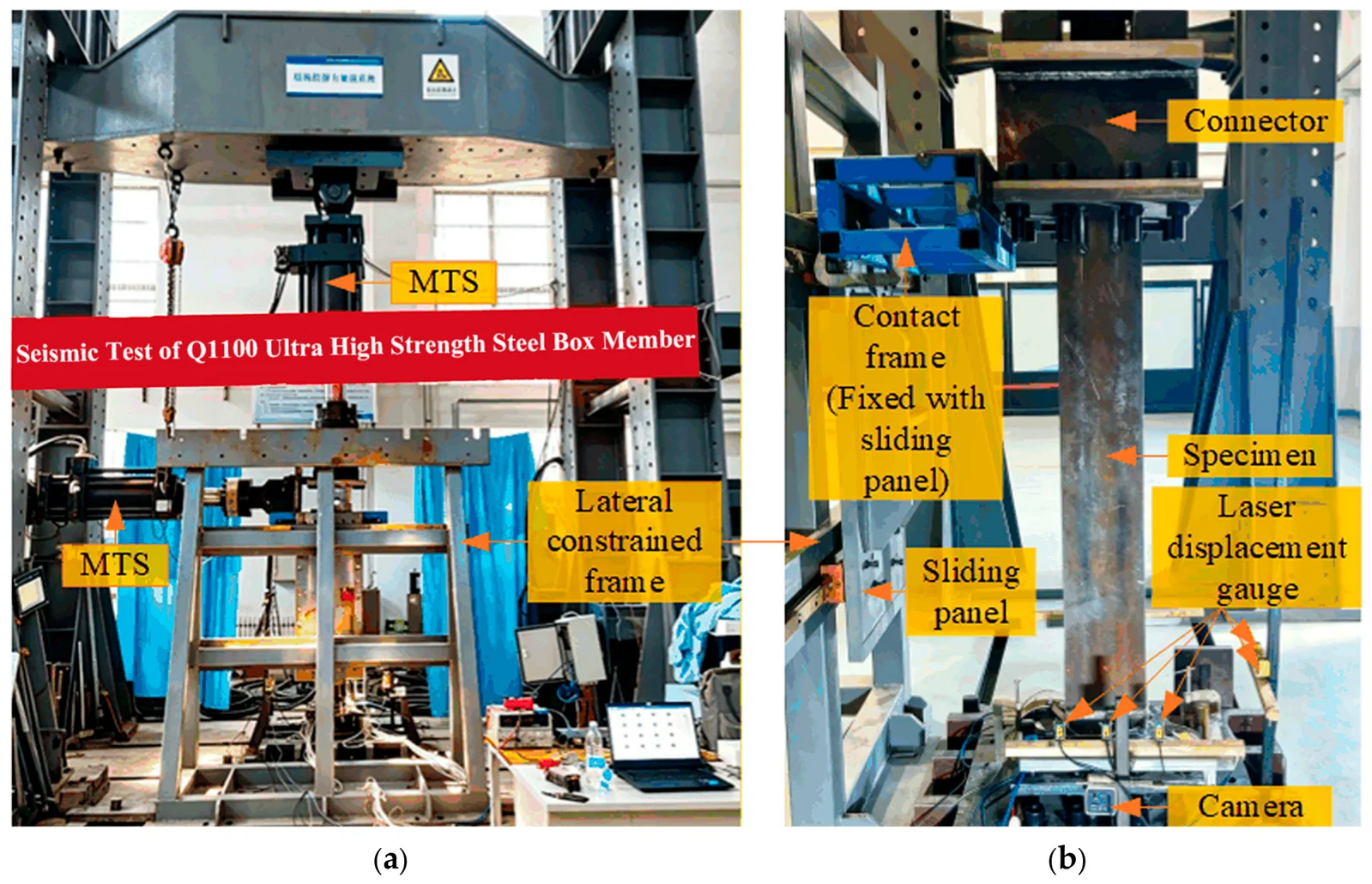
Schematic diagram of test setup: (**a**) frontal configuration; (**b**) lateral configurationfl.

All specimens were tested under combined constant axial compression and cyclic lateral loading. The axial load was applied and maintained constant throughout each test using a hydraulic jack (see Figure 3). The lateral cyclic loading was applied at the column top using a servo-hydraulic actuator under displacement control, following the loading protocol specified in JGJ/T 101 [31] or ATC-24 [32]. Lateral displacements were measured using draw-wire displacement sensors (ENLAI, ELH 200D/500, China) (ENLAI) at multiple heights along the column height. Strain gauges in Figure 3 were attached to the flanges and webs at critical locations to monitor local buckling initiation and progression.

All specimens were tested under the condition of top lateral free/top lateral contact constraint and the bottom fixed. Figure 3 shows a schematic diagram of the test setup. A rigid connection was established between the bottom end plate and the strong ground. A stiffened plate was installed at the top end (see Figure 3) to form a hinged connection, allowing the top end to rotate freely when axial and lateral forces were applied. The cyclic lateral force was generated by a 500 kN servo-controlled hydraulic actuator, and the constant axial force was applied by a 1000 kN vertical hydraulic jack (see Section 2.3.4). A sliding device was installed on the vertical jack to ensure the reliable existence of the constant axial force throughout the test. A lateral limiting system was used to restrict the out-of-plane displacement of the specimens.

**Figure 4:**
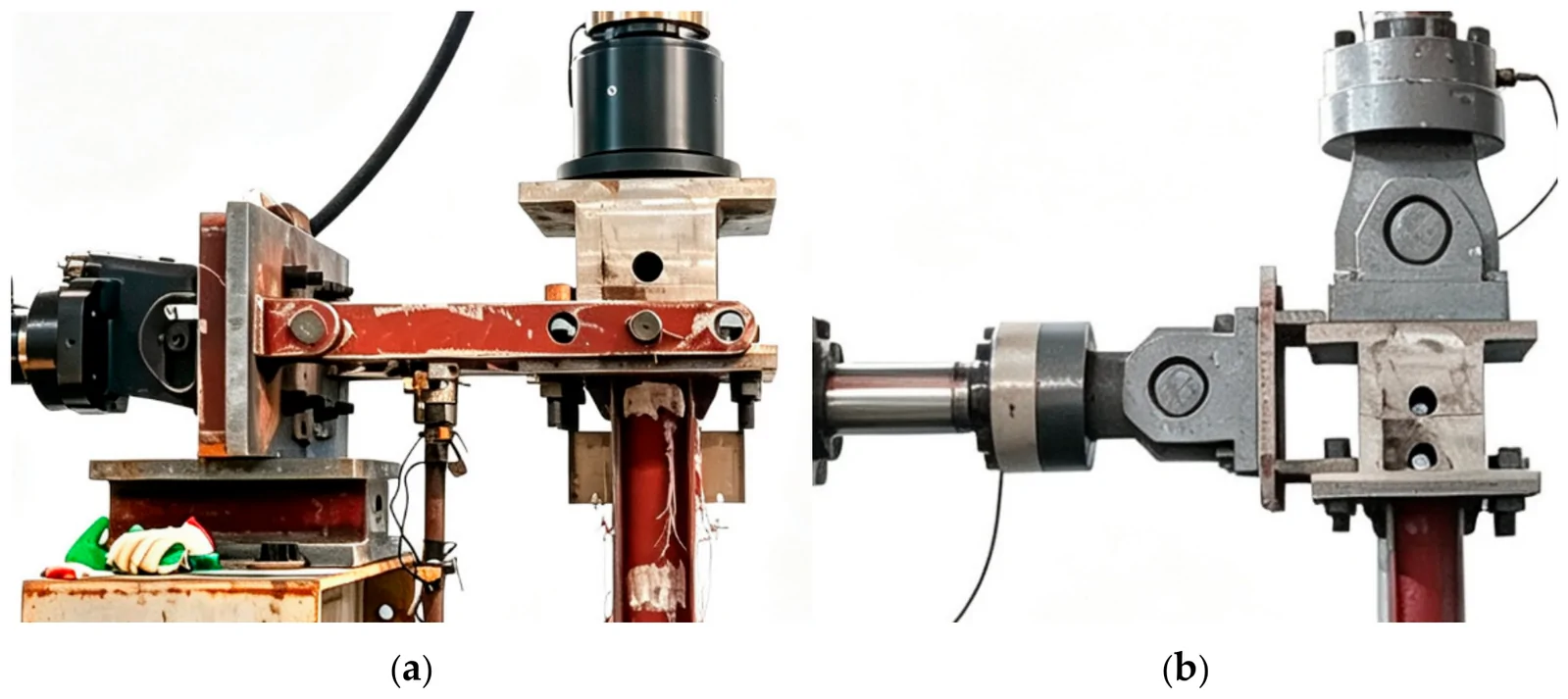
Schematic of the connection between the transverse MTS actuator and the specimen top: (**a**) hinged actuator end with sliding connection plate and parallel-link hinged joints; (**b**) hinged actuator end with fixed connection plate.

The fundamental difference between the parallel-link hinged connection in Figure 4a and the fixed connection in Figure 4b, both having a hinged MTS end, is that the former—introduces a sliding connection plate and hinged parallel links to allow additional relative movement and rotation, while the latter uses a rigidly fixed connection plate that suppresses such compliance, as illustrated in Figure 4.

#### 2.3.2. Lateral Support Conditions

Figure 3b presents the lateral boundary configuration. It illustrates the contact-constrained (C) case, i.e., the top-lateral-contact constraint setup. When the lateral constraint frame is removed, the setup becomes the unconfined (UC) case, i.e., the top-lateral-free, non-contact-constrained configuration. The constraint frame is equipped with a sliding plate. Under transverse MTS loading, once the specimen contacts the top lateral constraint, the sliding plate moves along the smooth guideway, resulting in negligible frictional resistance. Based on these configurations, the 10 numbered test specimen schemes fall into two distinct experimental groups.

The first group comprises all specimens with the suffix ‘UC’ (Nos. 1–7 and No. 10), which correspond—to the top-lateral-free setup, i.e., the non-contact-constrained case obtained after removing the lateral constraint frame. The second group consists of the two specimens with the suffix ‘C’ (Nos. 8 and 9), which belong to the top-lateral-contact constraint setup shown in Figure 3b.

#### 2.3.3. Instrumentation and Measurement

For each of the 10 test configurations, the surface displacements of the structural components were recorded with a laser displacement sensing system, which incorporated eight transducers positioned within an identical height range; the precise arrangement is depicted in Figure 3b. Three transducers were positioned on each flange side and two on the web side, resulting in a cumulative total of eight per test. Each transducer is a CMOS laser sensor (manufactured in China) with a peak output of 1 mW, with a maximum pulse duration of 5 ms, a wavelength of 650 nm, and an accuracy rating of 0.01 mm, capable of measuring displacements over a range of ±200 mm or more. The transducers measured out-of-plane panel displacements relative to the laboratory reference; the global column-top displacement was not subtracted. Local buckling was identified only where visual observation and residual differential deformation between the plate center and plate edges/stiffeners supported it.

#### 2.3.4. Data Acquisition System

Two pseudo-static testing systems were utilized in this experimental program, as indicated in Table 4. The first system consisted of a transverse MTS actuator (Model 244.41, MTS Systems Corporation, Eden Prairie, MN, USA; rated force 500 kN, a total stroke of 254 mm, and a maximum pressure of 20.7 MPa) alongside a vertical double-rod high-precision actuator (Model PWSL-1000, serial no. 202411011; Tianjin Weisi Experimental Instrument Co., Ltd., Tianjin, China, maximum force of 1000 kN, a maximum stroke of 200 mm and an accuracy class of 1). The transverse actuator was controlled through MTS 793 MultiPurpose TestWare^®^ running on a FlexTest digital controller (MTS Systems Corporation, Eden Prairie, MN, USA), whereas the vertical actuator employed a specialized data-acquisition system. The second system comprised a transverse MTS actuator (with a rated pressure of 21 MPa, static thrust of 1290 kN, and dynamic thrust of 870 kN, produced in January 2015) and a vertical MTS actuator (Model FCS211000-500, serial no. 201407-02-02; Beijing Foli System Co., Ltd., Beijing, China; rated pressure 21 MPa, static thrust of 2100 kN, dynamic thrust of 1600 kN, stroke of ±250 mm, overall length of 2575 mm, and width of 759 mm). On 3 April 2025, the vertical actuator of the first system malfunctioned after attaining the maximum load during Test No. 5, requiring a transition to the secondary system for subsequent testing. As a result, the initial four schemes (Nos. 1, 2, 3, and 5) were executed—using the first system, whereas the remaining six schemes were implemented with the second system. The vertical loading apparatus was adjusted prior to testing and demonstrated acceptable precision, with the average applied forces differing by no more than 0.8% from the intended values in all test scenarios.

To evaluate the comparability of test systems, Table 5 summarizes the vertical loading statistics for the selected physical members assigned to the two test systems.three For System 1, the 300 kN specimens (SA-Q1100) had large sample sizes (51,678 and 57,075) and mean forces of 297.957 and 299.380 kN, with deviations from the target of approximately 0.68% and 0.21%, respectively. For System 2, the 1450/1453 kN specimens (SB-Q1100 and SC-Q1100) also showed means close to their targets (1450.505 and 1451.475 kN; deviations ≈ 0.03% and 0.10%) despite markedly different standard deviations (6.935 and 1.520 kN). These results indicate that both systems achieved accurate loading under their respective target conditions and exhibited no evident systematic bias, supporting the reasonableness of using each system for its assigned tests. Nevertheless, the differing variability and system configurations mean that responses from the two systems are not treated as strictly equivalent; cross-system comparisons are therefore used only for qualitative trend and mechanism assessment.

### 2.4. Quasi-Static Cyclic Loading Protocol

**Figure 5:**
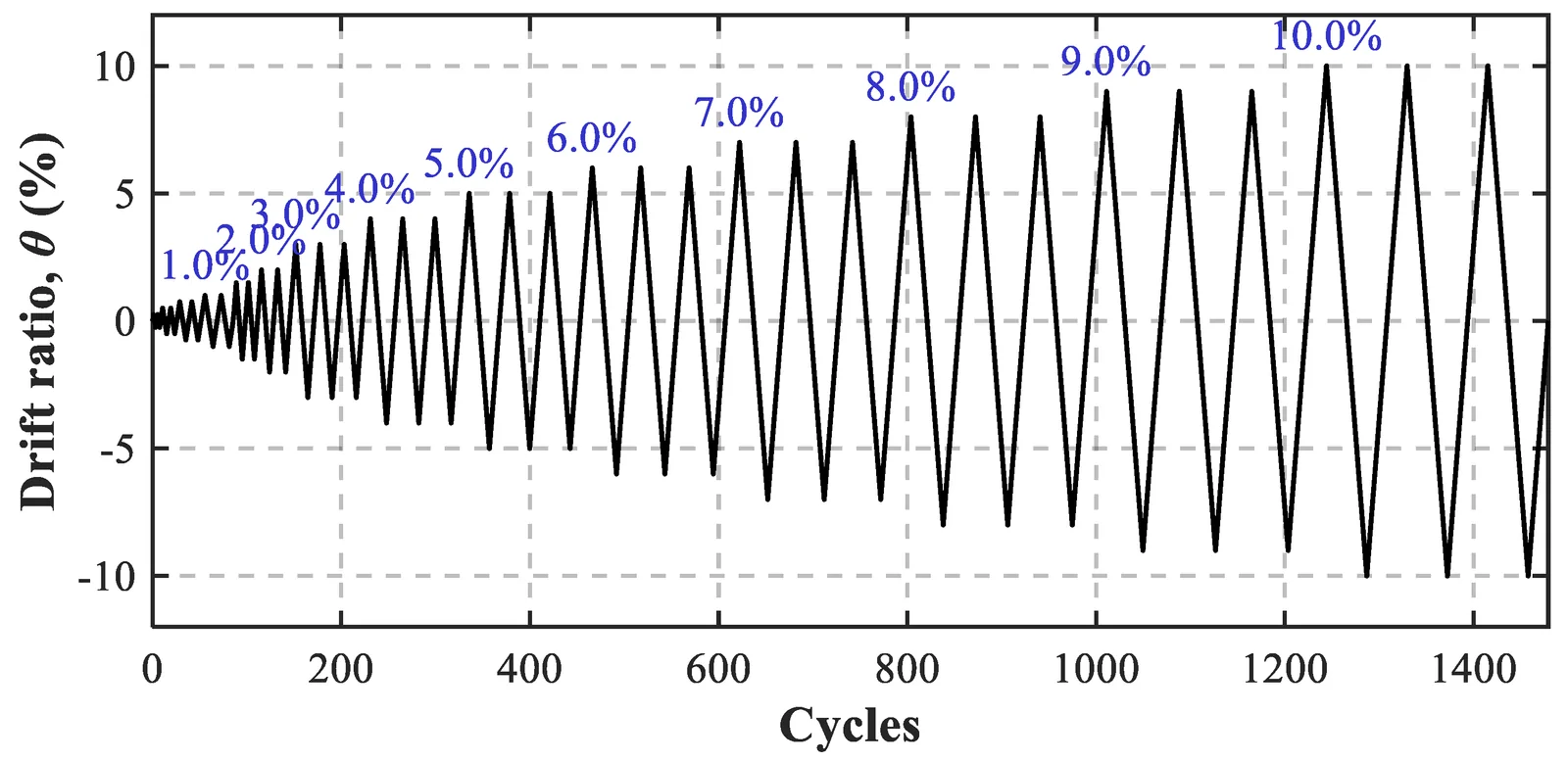
Cyclic loading protocol (displacement-controlled).

The quasi-static cyclic loading protocol was displacement-controlled and applied in successive stages, as shown in Figure 5. The loading programme comprised 14 amplitude levels, corresponding to target inter-storey drift ratios (DR) from 0.25% to 10.00%, featuring incremental increases of 0.25% for the initial four levels (0.25–1.00%) and then bigger increments up to 10.00%. For levels not exceeding 1.00%, a uniform loading rate of 1 mm/s was employed, with each level being replicated for two complete reversed cycles. For drift levels from 1.50% to 10.00%, the loading rate was increased to 2 mm/s in each cycle. Tests were terminated at 10% drift or when notable strength deterioration was visually observed; no quantitative termination threshold was recorded. Every cycle exhibited a symmetric triangle waveform, commencing at zero, ascending to the positive apex, descending back to zero, dipping to the negative trough, and ultimately returning to zero. The maximum displacement *Δ_p_* at every level was determined by multiplying the designated drift ratio by the specimen height *H*; for instance, with *H* = 1710 mm, *Δ_p_* equals 4.275 mm for a drift of 0.25%. Each test was terminated on reaching the 10% drift threshold or when marked strength deterioration was observed. This loading timetable conforms to conventional guidelines for assessing seismic performance of structural elements subjected to significant inelastic deformations.

### 2.5. Section Classification According to Width-to-Thickness Ratios

The local stability of each plate was assessed using the limiting width-to-thickness ratios outlined in the Chinese standard GB 50017 [30] and JGJ/T 483-2020 [14]. The dimensionless variable *ε* is expressed as $\varepsilon =\sqrt{235/f_{y}}$. For Q1100 steel, $\varepsilon =\sqrt{235/1210}=0.441$; therefore, the Class 3 threshold for extended (unsupported) flanges is 14*ε* = 6.17. According to the dimensional configuration depicted in Figure 1, the width-to-thickness ratios of the internal flange and the extended flanges [14] can be determined. The internal flange (linking the web) exhibits a ratio of *b*_0_/*t_f_* = 53/8 = 6.63, far lower than the Class 1 internal-element threshold of 33*ε* = 14.54. As a result, the internal web-flange connection is categorized as a Class 1 (plastic) portion for all samples. The extended flange plates in the SB and SC sections have *b/t* ratios of 6.875 (for 55 mm plates) and as high as 13.125 (for the 105 mm plate). Given that these values significantly surpass the Class 3 limit of 6.17, these extended-flange plates are designated as Class 4 (slender) components for all Q1100-grade specimens. This suggests that the local plate buckling of the reinforcing components will dictate the ultimate strength before achieving complete plastic cross-sectional resistance; it does not by itself define the member failure mode. In the case of the solitary Q345 specimen (SB-Q345-InsWeld-DBS-455-C), with *ε* = 0.825 (Class 3 threshold = 11.55), the extended plate (*b*/*t* = 6.875) is categorized as Class 3.

### 2.6. Effective Lengths, Slenderness Ratios, and Axial Load Levels

All specimens were tested under monotonically applied a constant axial compression using the vertical actuator and the cyclic load by the transverse actuator coupled with a linkage system. The first four ‘UC’ (unbraced) specimens adopted an effective buckling length of *L*_eff_ = 1710 mm, while the remaining six specimens were assigned *L*_eff_ = 1845 mm based on the specific end-fixture detailing and actuator stroke. For the ‘UC’ boundary condition, no lateral bracing is provided about the minor axis; hence, the minor-axis radius of gyration (*r_y_*) governs the flexural buckling resistance. For the two laterally braced (‘C’) cases, the minor axis is restrained, and the major-axis radius (*r_x_*) becomes the governing parameter. The resulting slenderness ratios (*λ* = *L*_eff_/*r*) range from 30.75 (stocky braced specimens) to 100.98 (slender unbraced SB specimens), covering a broad spectrum of column behaviors.

The targeted axial compressive loads (*N*) were chosen to examine the inelastic behavior throughout several levels of compression dominance, specifically 300 kN, 455 kN, 800 kN, 1030 kN, 1450 kN, and 1453 kN. To standardize the exerted loads relative to the material’s capacity, the axial load ratio is expressed as *n* = *N*/(*A* × *f_y_*), with *A* representing the gross cross-sectional area and *f_y_* denoting the actual yield strength. As outlined in Table 6, the axial load ratios vary from 0.057 (lightly loaded SA specimens) to 0.216 (Q345 braced specimen), facilitating a comprehensive assessment of the interrelated impacts of local slenderness, global slenderness, and compression intensity on the structural efficacy of built-up ultra-high-strength steel columns.

## 3. Experimental Results

### 3.1. Failure Modes and Damage Evolution

#### 3.1.1. Test Observations and Failure Modes

**Figure 6:**
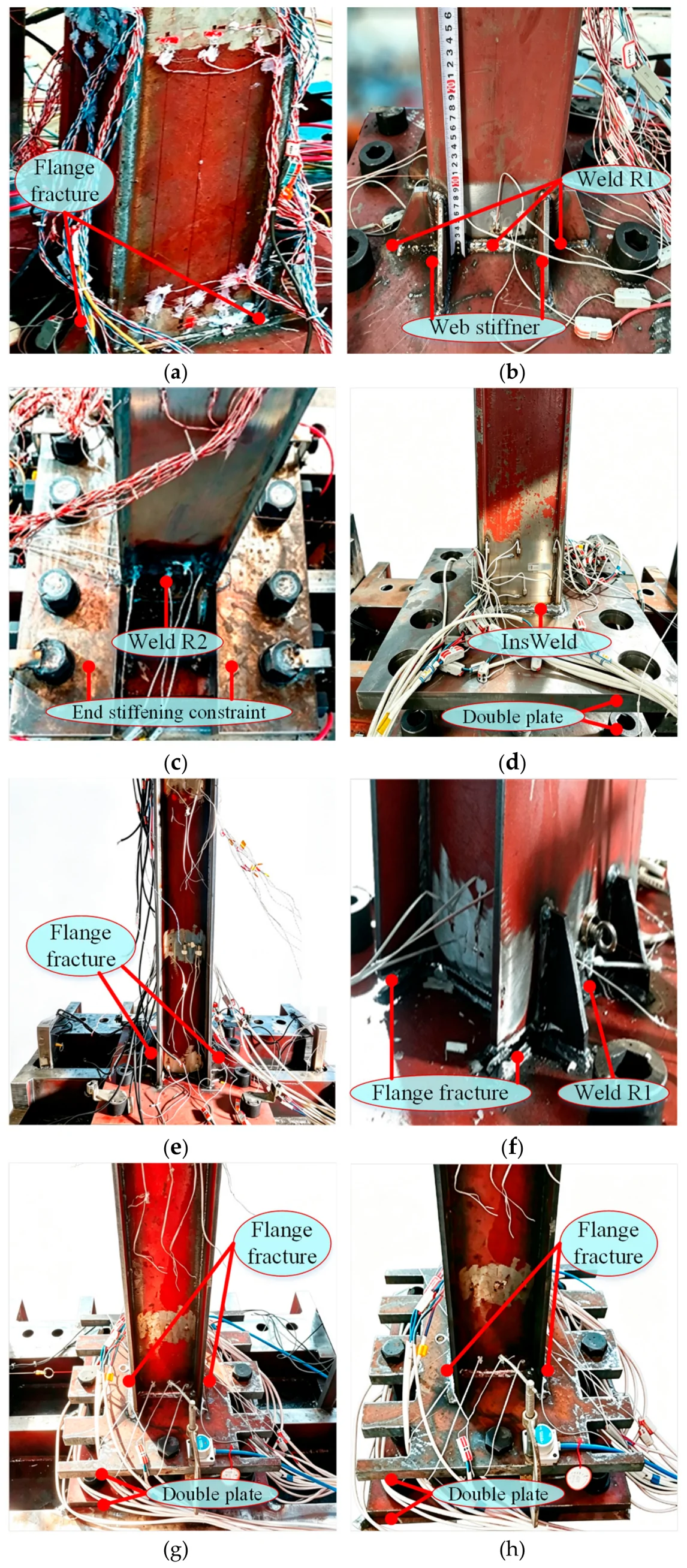

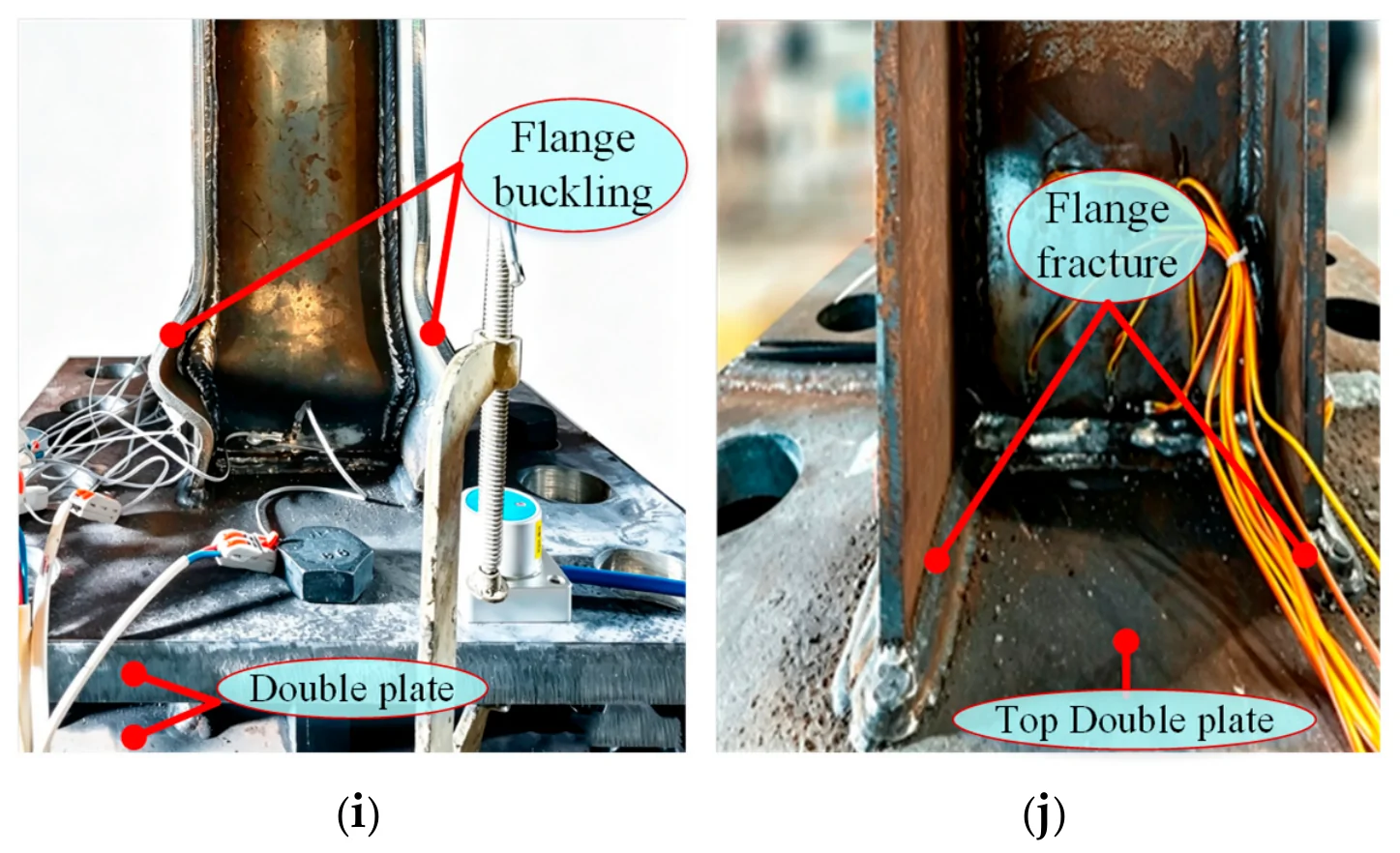
Failure modes of all specimen schemes: (**a**) No. 1; (**b**) No. 2; (**c**) No. 3; (**d**) No. 4; (**e**) No. 5; (**f**) No. 6; (**g**) No. 7; (**h**) No. 8; (**i**) No. 9; (**j**) No. 10.

In the Q1100 specimens (Figure 6a–h,j), fractures consistently initiated at the lower plate or weld region and then propagated from the flange into the web. Local buckling of the bottom plate manifested in some large-amplitude cycles but rectified after reversal; this was consistent even for the wider-flange SC specimen depicted in Figure 6j, the Q345 stiffening ribs experienced localized buckling under constraint; nevertheless, the ultimate failure remained fracture-controlled due to the predominance of the Q1100 base metal and WAZ.

The SB-Q345 specimen depicted in Figure 6i adhered to an alternative trajectory. Localized severe buckling initiated at the lower edge of the protruding flange, subsequently propagating to the intermediate flange and web as the vertical stress persisted. This gradual deformation ruptured the flange-web weld, leading to the eventual collapse of the buckled plate segments into a single plane, resulting in total loss of load-bearing capacity.

These two patterns signify the substantive distinction in observed failure mode. Q1100 exhibits considerable strength but restricted member-level deformation capacity. WAZ embrittlement is a possible explanation; other variables such as axial load ratio and section geometry also differ between the Q1100 and Q345 specimens.

#### 3.1.2. Out-of-Plane Displacement of Steel Flange and Web Panels

Out-of-plane displacement data from four box-section specimens were analyzed numerically (Nos. 4, 8, 9, and 10). The unprocessed data comprised eight channels in Figure 3b: two flange planes (three each) and a single web plane. Web anomalies resulting from signal interruptions were identified by first-order differentiation employing median and MAD; deviations exceeding 5 MAD were substituted with NaNs and subsequently linearly interpolated. Flange groups were categorized as “wide” or “narrow” based on the overall displacement range (maximum minus minimum). Time was standardized to the interval [0, 1], all signals were interpolated to 2000 shared points, and six statistics (initial, final, maximum, minimum, range, and standard deviation) were derived—for each component (wide flange, narrow flange, web). Raw and filtered comparisons and sensitivity checks for the 5-MAD threshold were not archived; the filtering was intended only to remove signal interruptions, not to identify local buckling. The “wide/narrow” labels therefore refer to sensor range, not to a code-based slenderness classification.

**Figure 7:**
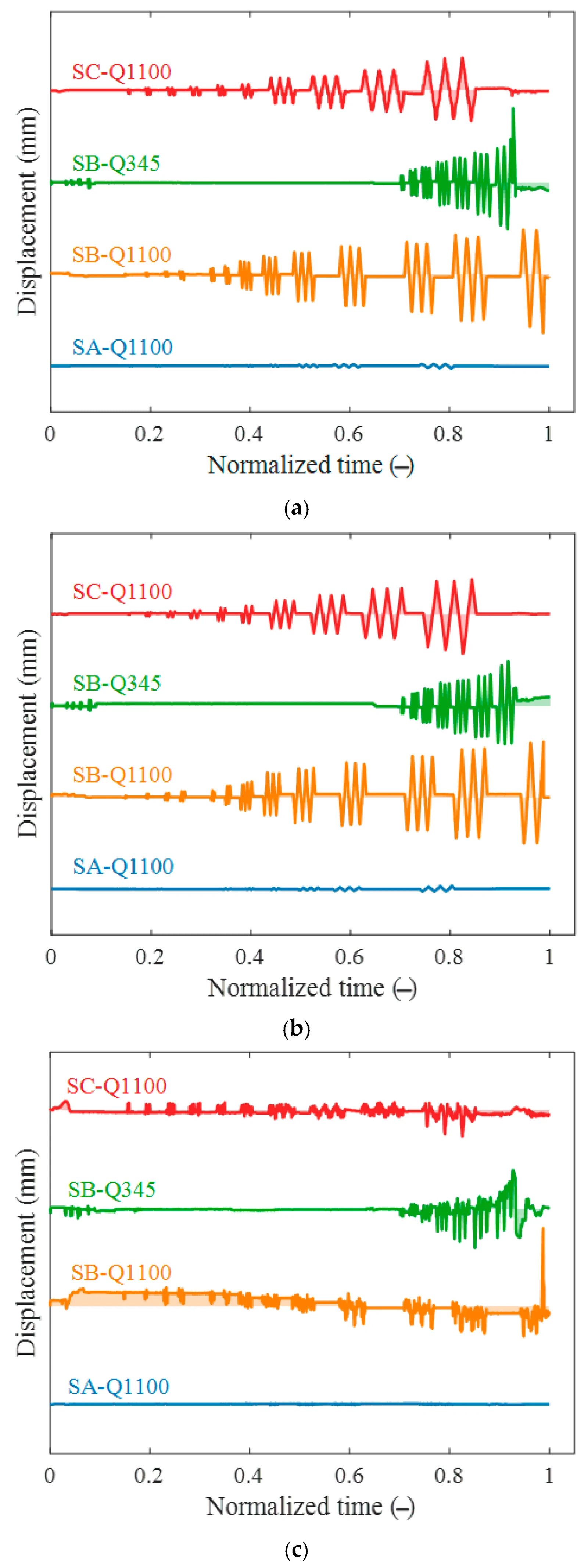
Out-of-plane displacement comparison among 4 schemes (including SA-Q1100-InsWeld DBS-1030-UC, SB-Q1100-InsWeld DBS-1450-C, SB-Q345-InsWeld DBS-455-C, SC-Q1100-InsWeld DBS-1453-UC): (**a**) wide-flange mean displacements; (**b**) narrow-flange mean displacements; (**c**) web mean displacementswnw (with outlier removal).

Figure 7 compares the out-of-plane displacements of four configurations: (a) wide flange, (b) narrow flange, and (c) web (excluding outliers). SA-Q1100 maintained a virtually elastic state (flange approximately 1.69 mm; web 0.16 mm). SB-Q1100 exhibited the largest out-of-plane flange displacement, with a flange measuring up to 37.7 mm, a web of 10.2 mm, and a standard deviation of 4.39. SB-Q345 exhibited the most extensive wide-flange range (44.2 mm) while maintaining a consistent web displacement (7.3 mm), suggesting flange yielding instead of web failure. SC-Q1100 exhibited moderate flange displacement (about 23 mm) and a consistent web displacement (3.34 mm). Web displacements were invariably far less than flange displacements. Residual displacements remained around 1.4 mm, with the exception of SB-Q345 at approximately 3.0 mm, indicating localized plastic buckling in Q345; Q1100 exhibited no significant local buckling. The most significant flange movement measured was 27.0 mm in SB-Q345. The flange displacements of SA-Q1100 and SC-Q1100 were less than those of SB-Q1100, mostly due to a lower axial load ratio (SC 0.174 compared to SB 0.196) and drift (5–6% against 9%), which mitigated *P*-*Δ* effects and delayed flange out-of-plane deformation. These values are out-of-plane panel displacements relative to the laboratory reference; the global column-top displacement was not subtracted. Local buckling was identified only where visual observation and residual differential deformation supported it.

### 3.2. Hysteretic Response

#### 3.2.1. Hysteresis Loops of SA-Q1100 Series

**Figure 8:**
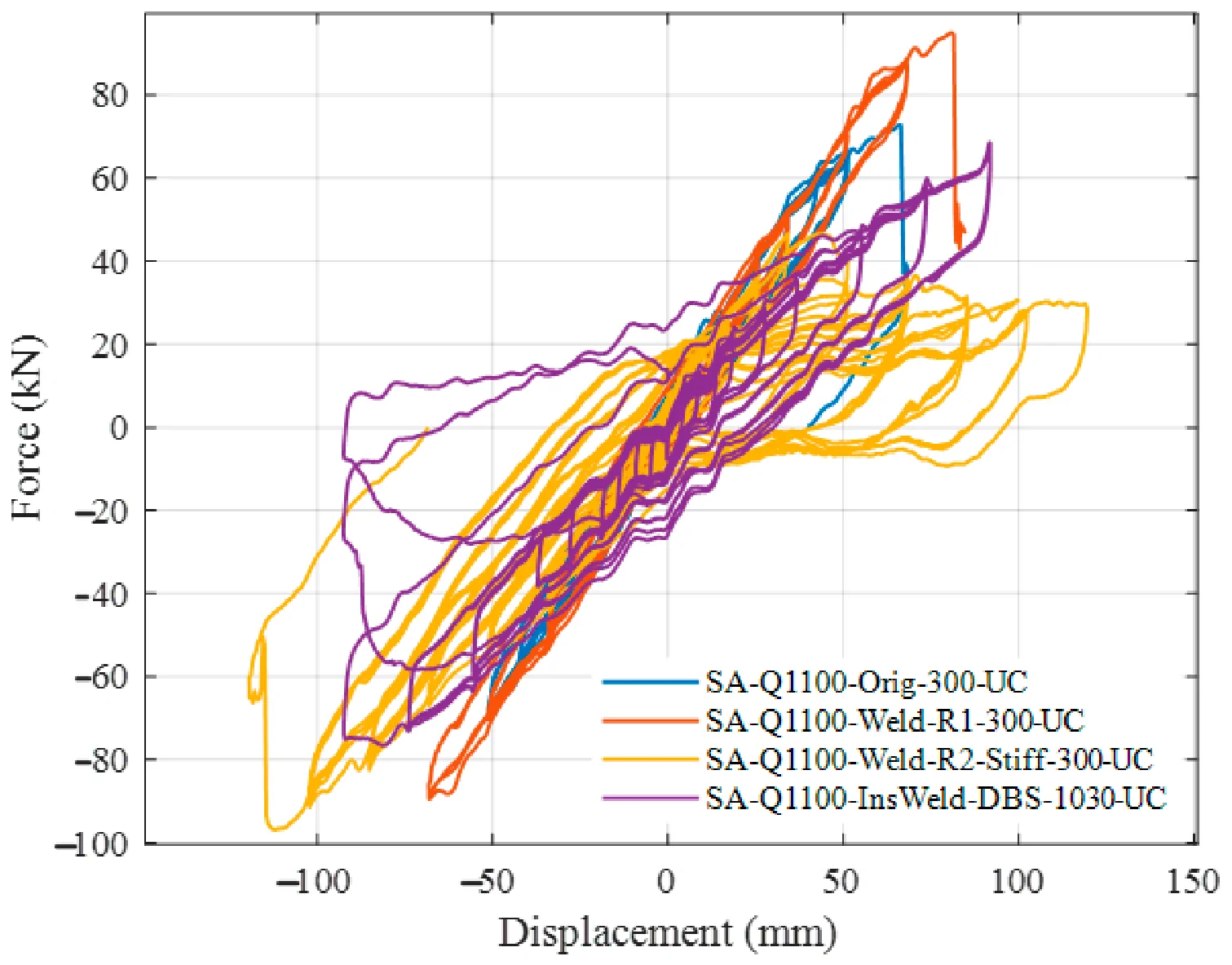
Hysteresis loops of SA group.

Figure 8 illustrates that the hysteretic characteristics of SA-Q1100 extended box-section flange specimens progressively decline with an increasing number of repair-weld passes. Under the initial and first-repair circumstances, the response was predominantly linear-elastic, exhibiting almost symmetrical, spindle-shaped hysteresis loops; the first repair marginally enhanced both load-bearing capacity and deformability. Following the second reinforcement with stiffeners, significant pinching and a decline in stiffness were seen in the positive loading direction, whereas the negative direction exhibited almost linear-elastic behavior, suggesting a decoupling of energy dissipation between tension and compression. Despite the final displacement surpassing 110 mm (DR = 6%), the descending branches grew more abrupt. Following the third repair, which included plug-welded end plates and double bottom plates subjected to increased axial stress, the load-bearing capacity failed to rebound; the behavior displayed brittle post-peak softening and significant pinching in the negative direction, accompanied by a notable decrease in energy dissipation.

The hysteresis loops transitioned from symmetrical and robust to asymmetrical and constricted, finally culminating in brittle fracture in the negative direction. This morphological pathway is characterized by reduced loop areas, heightened constriction, more abrupt descending limbs, and separated positive/negative energy dissipation. These observations are consistent with, but do not establish, an accumulation of damage in the WAZ associated with successive welding thermal cycles, which may involve grain coarsening, steeper hardness gradients, and additional residual stresses; these mechanisms were not directly characterized in this study, and the axial load was also higher in No. 4. Accordingly, the asymmetry is reported here only as a direction-dependent response tendency of the WAZ rather than as a demonstrated directional degradation mechanism, and no causal attribution is implied. The fFinal fracture was consistently located at the lower plate/weld region within the WAZ, rather than in the parent material or stiffener core.

#### 3.2.2. Hysteresis Loops of SB-Q1100 and SB-Q345 Series

**Figure 9:**
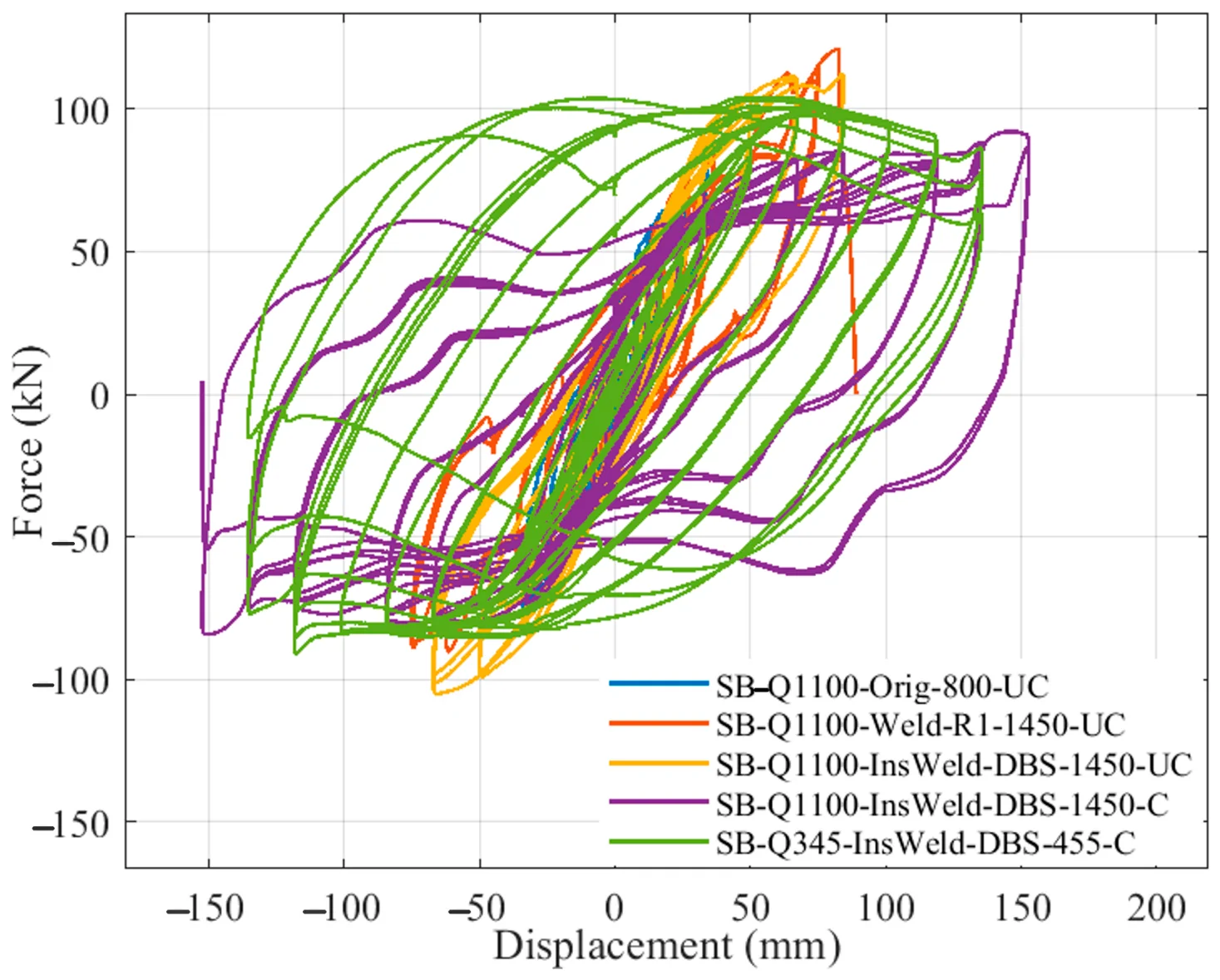
Hysteresis loops of SB group.

Figure 9 compares the hysteretic behaviors of SB-Q1100 and SB-Q345 specimens across several repair scenarios. The initial SB-Q1100 sample displayed robust, almost antisymmetric spindle-like loops but succumbed to bottom-weld fracture at approximately 2% DR, suggesting restricted deformability. Following the initial repair, capacity was reinstated and deformability enhanced (DR ~5%), with no noticeable pinching. The second repair (plug welding + double bottom plates) resulted in increased loop areas, partial restoration of capacity, and a DR of around 7%, accompanied by a minor decline in energy efficiency. The third modification, which involved incorporating upper lateral contact limitations, enhanced loop fullness, capacity, and maximum displacement (DR ~9%); the responses, both positive and negative, were nearly symmetrical. Local curve fluctuations resulted from the movement of the lateral constraints, rather than from structural instability. Under the same specifications, SB-Q345 demonstrated more pronounced loops compared to SB-Q1100, consistent with the enhanced plastic energy absorption of lower-strength steel. In general, the capacity in the positive direction had a minor decline with repair passes, while the capacity in the negative direction diminished following the initial repair and somewhat rebounded after the second. This uneven progression is consistent with a direction-dependent response; however, its cause is unresolved, and other parameters, including axial load, also differ.

#### 3.2.3. Hysteresis Loops of SC-Q1100 Series

**Figure 10:**
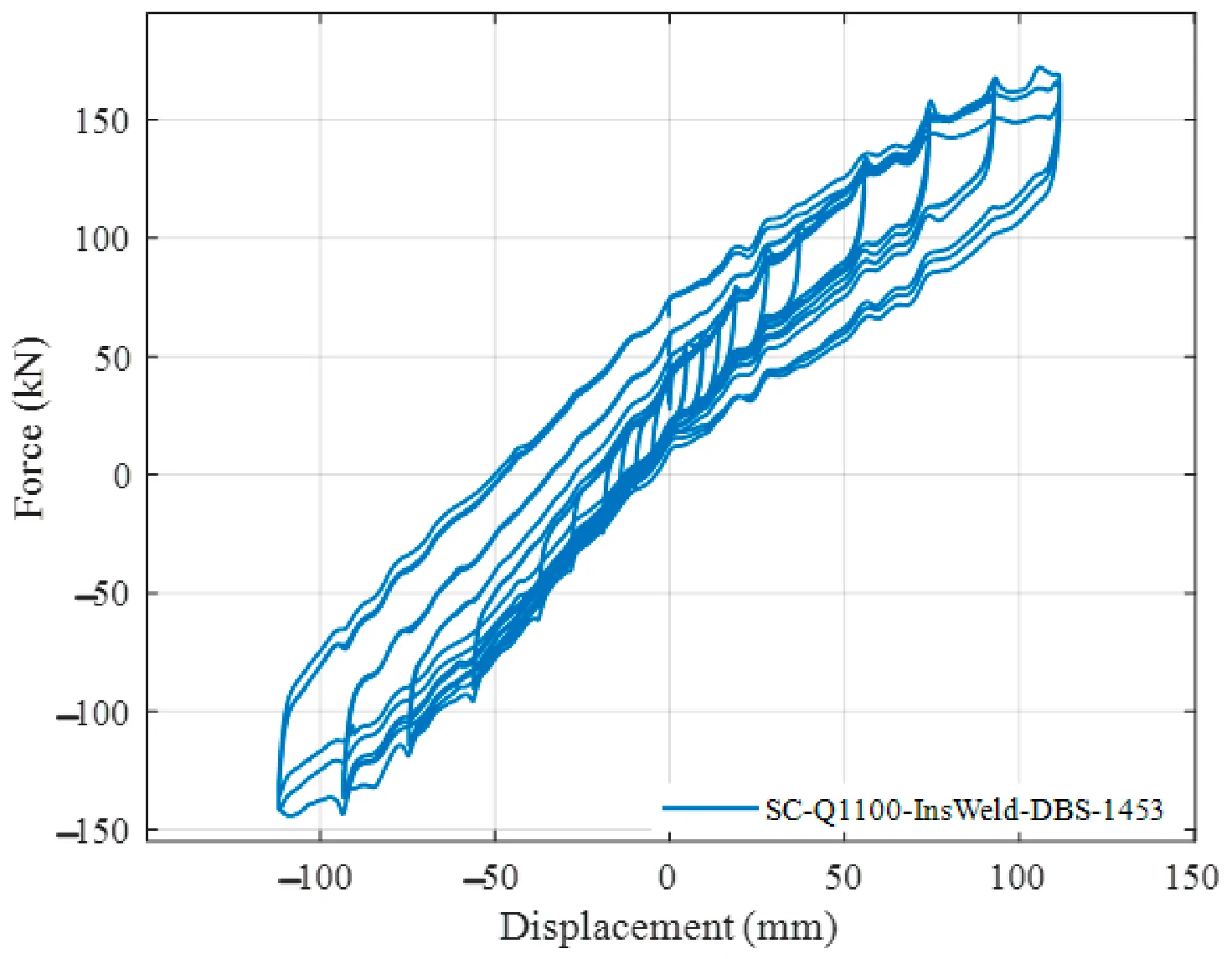
Hysteresis loops of SC specimen.

Figure 10 illustrates the hysteretic characteristics of SC-Q1100 specimens, including non-uniform-width extended flanges subsequent to the plug-weld restoration of the end plate and the incorporation of twin bottom plates. An initial lateral load offset of about 27.93 kN arose from a centroid eccentricity under the 1453 kN vertical load. Upon eliminating this offset, the hysteresis loops exhibit a flattened spindle configuration, devoid of notable pinching or deterioration in stiffness, signifying gradual joint impairment. The load in the positive direction is marginally greater than that in the negative direction, with both ultimate displacements above 110 mm (DR = 6%). The somewhat reduced positive slope indicates a modest deterioration of positive-direction energy, perhaps resulting from deformation concentration caused by the broader flange. In general, the repair successfully restored capacity, deformability, and energy dissipation, mitigating pinching and postponing stiffness deterioration. In practice, this initial load offset should be eliminated by adjusting the loading alignment. In contrast to SA specimens, SC specimens exhibit more significant positive-negative discrepancies due to increased cross-sectional asymmetry; thus, repair design must include cross-sectional configuration.

### 3.3. Skeleton Curves and Envelope Response

**Figure 11:**
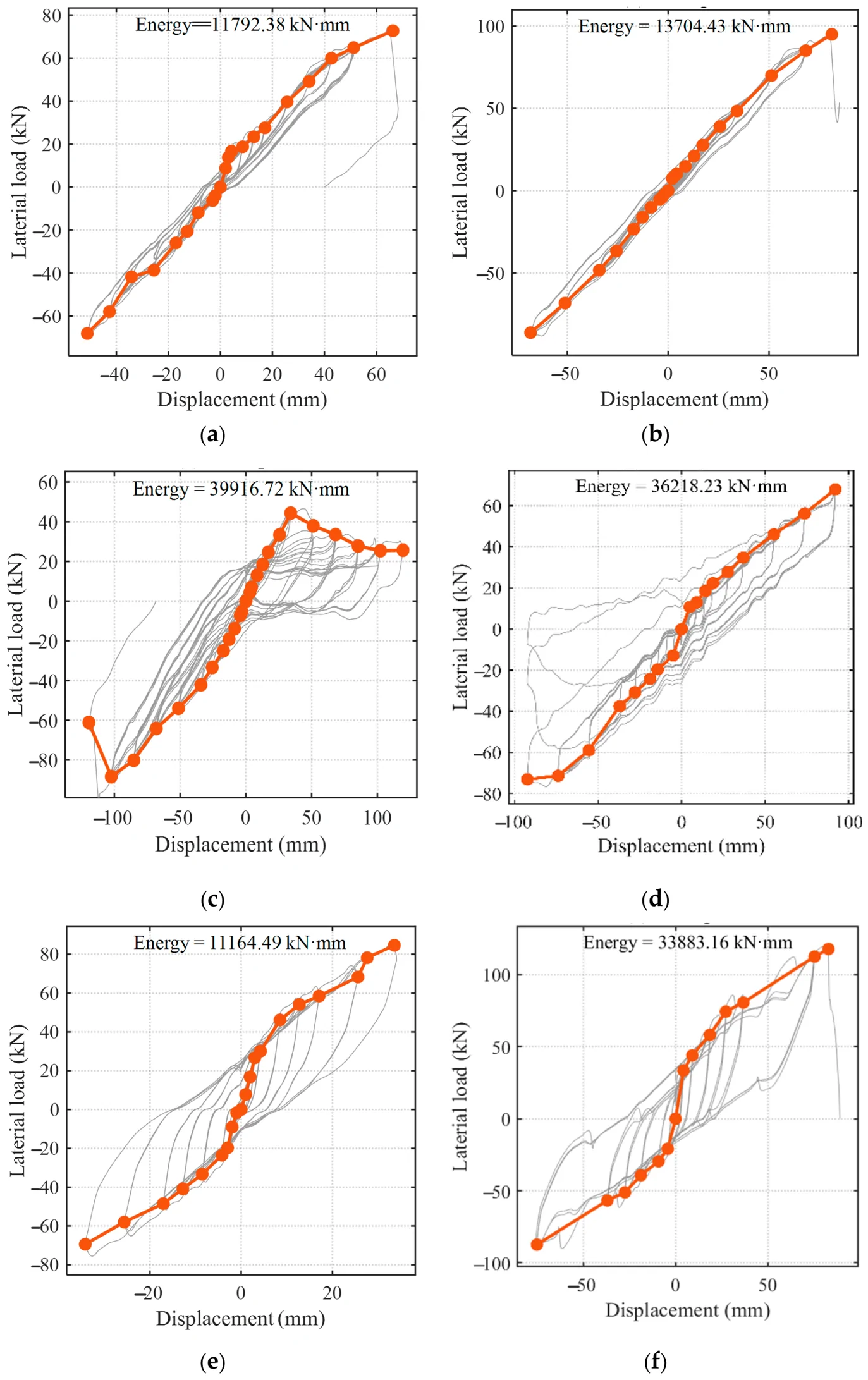

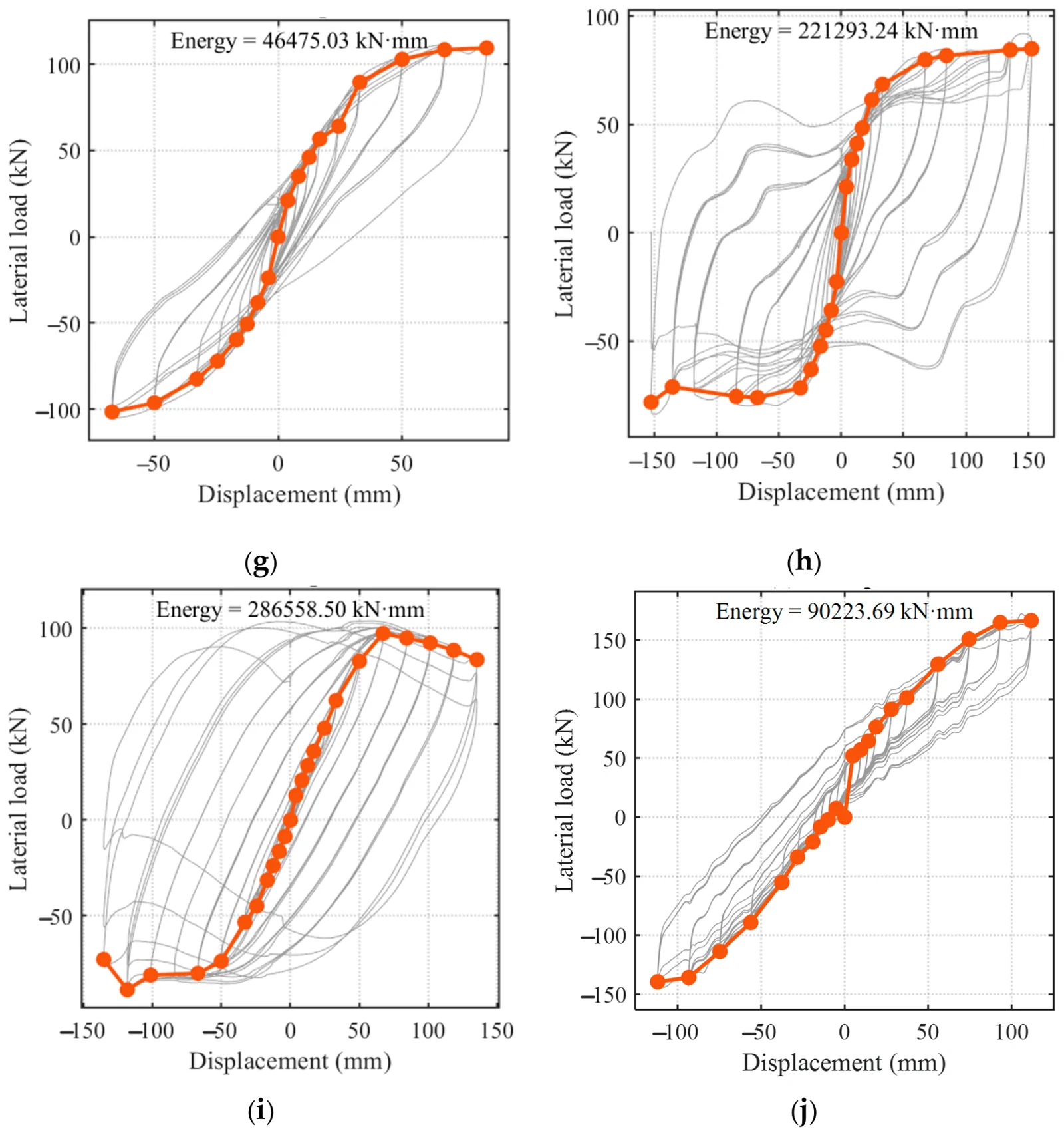
Skeleton curves and envelope responsecer: (**a**) No. 1; (**b**) No. 2; (**c**) No. 3; (**d**) No. 4; (**e**) No. 5; (**f**) No. 6; (**g**) No. 7; (**h**) No. 8; (**i**) No. 9; (**j**) No. 10.

Figure 11 delineates the skeleton curves with the peak-point technique, revealing significant morphological variation among the specimens.

Nos. 1 and 2 (SA-Q1100, original and first repair) both displayed nearly linear responses with small loops; No. 1 suffered a brittle weld fracture before yielding, whereas No. 2 showed limited WAZ damage and a slightly higher capacity. No. 3, following a secondary repair using stiffeners, exhibited a downward trend due to cracking in the positive-direction flange, signifying a reduction in strength. No. 4, exhibiting a high axial ratio (0.195) following a third repair, demonstrated early hardening/brittleness and a swift decline after peak performance, maintaining a nearly linear trajectory with a minor loop despite the presence of double bottom plates. In the SB series, No. 5 (axial ratio 0.108) exhibited an S-shaped framework characterized by a yield plateau and a rather substantial loop, but its plasticity was constrained (DR 2%). Nos. 6 and 7, following repeated repairs with double bottom plates, displayed increasingly pronounced S-curves and expanded loops (DR 5% and 7%, respectively). No. 8, constrained laterally at the top, attained a robust S-shape and DR 9%, the highest among SB-Q1100. No. 9 (Q345) produced the most pronounced S-shape and largest loop, signifying enhanced plastic dissipation. No. 10 (SC-Q1100) had an asymmetric profile that produced an initial load offset; its rectified framework was a flattened spindle/nearly linear shape with a minor loop, primarily influenced by elastic bending.

In summary, the shape of the skeleton is associated with material yield properties, axial load ratios, possible cumulative WAZ effects, external constraints, and cross-sectional asymmetry. Elevated axial stress, robust Q1100 steel, and possible thermal impairment may inhibit plastic formation, resulting in linear or softened frameworks. Low-strength steel, efficient upper lateral restraints, and well-organized repairs promote complete S-shaped curves and enhanced energy dissipation. The SA series responds in a non-linear manner, the SB series becomes progressively fuller as strengthening is added, and the SC series shows a nearly linear response with an initial offset that has to be corrected in evaluation and design.

### 3.4. Basic Performance Indicators

In this paper, the ultimate drift (DR) is defined operationally as the drift at test termination. It is not equated with inherent plastic-rotation capacity, because plastic rotation was not separately measured and no independent failure threshold was defined. The DR values presented in Table 7 exhibit numerous distinct patterns. For items 1 to 3 (SA), the axial load ratio is constant at 0.057, whereas the drift ratio increases from 4% to 5% and then to 6% as the repair process intensifies (original, first repair, second repair with stiffeners). Under identical normalized loading conditions, the use of repetitive repair welding and supplementary stiffeners did not diminish the post-yield deformation reserve; rather, it augmented it. No. 4 is more indicative: its load ratio stands at 0.195, approximately 3.4 times greater than that of Number 2, although its DR remains at 5%, the same as that of No. 2. This indicates that the incorporation of end welding along with twin bottom plates might enhance both strength and deformation compatibility.

In the SB group (A = 6120 mm^2^), specimens No. 7 (Q1100, *μ* = 0.196) and No. 9 (Q345, *μ* = 0.216) exhibit comparable load ratios, with DR values of 7% and 8%, respectively. The somewhat elevated DR of No. 9 suggests superior intrinsic ductility of Q345, but No. 7 bears over threefold the absolute load with merely a small reduction in deformability. The comparison of constraint is evident: with identical load, material, and load ratio (*μ* = 0.196), top-free No. 7 yields a DR of 7%, while top laterally constrained No. 8 attains 9%. Lateral end restraint was associated with enhanced axial displacement capacity, presumably by postponing local buckling.

A comparison of No. 7 and No. 10, both Q1100 featuring inserted end welding and twin bottom plates, reveals the influence of section dimensions despite their similar absolute loads (1450 kN against 1453 kN). The more extensive SC segment of No. 10 exhibits a diminished load ratio (0.174 compared to 0.196) and a marginally reduced DR (6% vs. 7%). For a specific absolute load, an increased cross-sectional area diminishes the stress intensity and lessens the observed axial deformation. This must not be mistaken for an authentic size-related reduction in ductility.

Table 7 presents a comparison of the essential parameters and ultimate drift ratios for ten experimental designs.

### 3.5. Energy Dissipation Capacity

**Figure 12:**
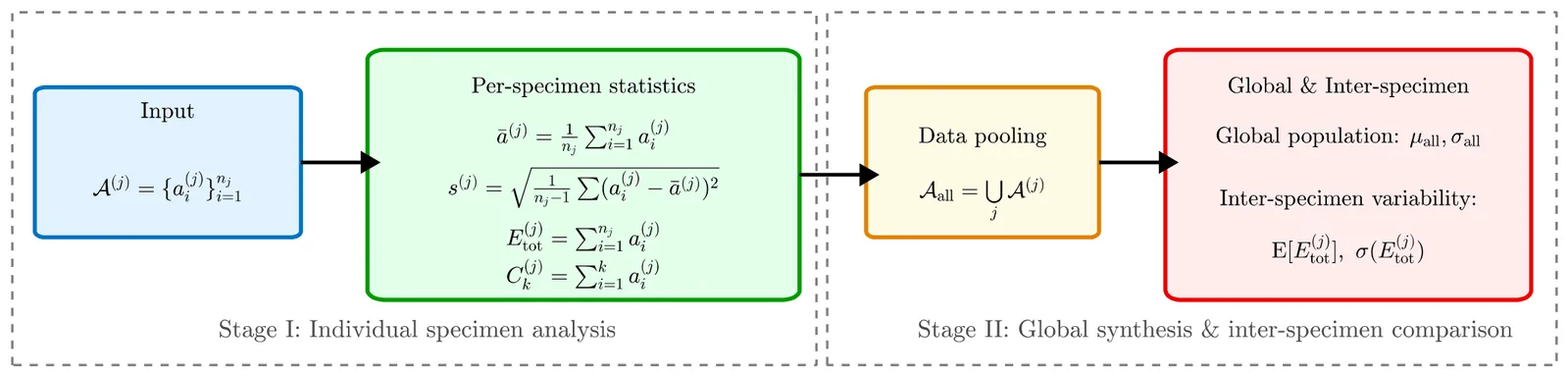
Computational framework for hysteretic energy dissipation data reduction.

A statistical framework shown in Figure 12, following the cyclic test procedure of ASTM E2126-19 [33], was established to evaluate hysteretic energy dissipation across test schemes. For each specimen, the force–displacement response gives a sequence of per-cycle dissipated energy values. For each complete cycle, the dissipated energy was computed by trapezoidal integration of the corrected lateral force–displacement data:

(1) $$E_{i}=\sum_{k}\frac{1}{2}(F_{k}+F_{k+1})(\delta_{k+1}-\delta_{k})$$

where *F_k_* and *δ_k_* are the force and displacement at consecutive data points, and the integration is taken around one complete positive–negative excursion using the trapezoidal rule [34]. The force baseline was zeroed using the initial unloaded reading; the displacement baseline was not shifted because actuator displacement was used as the reference. Incomplete cycles and cycles interrupted by signal loss were excluded. An illustrative calculation is not reproduced here; the integration script and raw records are retained by the corresponding author. From the per-cycle energy sequence, cumulative energy, mean, standard deviation, coefficient of variation, minimum, maximum, median, range, and progressive sum were calculated. All per-cycle data were then pooled to obtain global statistics, including grand total energy, global mean, and overall standard deviation. Finally, the mean, standard deviation, minimum, and maximum of the per-specimen total energies were computed to describe variability across the test matrix. No repeated tests under identical conditions were performed; therefore, these statistics do not establish reproducibility.

The cyclic test results, including the number of cycles to failure and the total dissipated energy, are compiled for all ten schemes in Table 8. Within the unconfined (UC) category, No. 10 (SC-Q1100-InsWeld-DBS-1453-UC) exhibits the highest total dissipated energy (88,686 kN·mm) after 23 cycles, whereas No. 5 (SB-Q1100-Orig-800-UC) exhibits the lowest (11,091 kN·mm) after 9 cycles. These total values are influenced by the different cycle numbers and imposed amplitudes. The two laterally restricted (C) specimens, No. 8 (SB-Q1100-InsWeld-DBS-1450-C) and No. 9 (SB-Q345-InsWeld-DBS-455-C), show higher total energy (227,786 and 285,545 kN·mm) after 25 and 29 cycles, respectively. However, their cycle counts are not disproportionately higher than those of the UC specimens (9–25 cycles); therefore, the higher total energy cannot be attributed to cycle count alone and should not be interpreted as equal-amplitude superiority. The higher total energy in the laterally constrained specimens is consistent with, but not attributable solely to, lateral contact restraint, because cycle count and other parameters also differ. Moreover, within the UC schemes, repair welding or reinforcement was generally associated with enhanced energy dissipation relative to the as-received state, but the extent of enhancement varied with geometric and material characteristics. The higher total energy of the Q345 specimen (No. 9) than that of the SB-Q1100-C specimen (No. 8) should not be attributed solely to material grade, because axial load ratio, section dimensions, and other parameters also differ. The cycle counts in Table 8 are reported as recorded and are not normalized for equal-amplitude comparison.

## 4. Discussion

### 4.1. Failure Mode Transition Under Multiple Welding Repairs and Strengthening Measures

**Figure 13:**
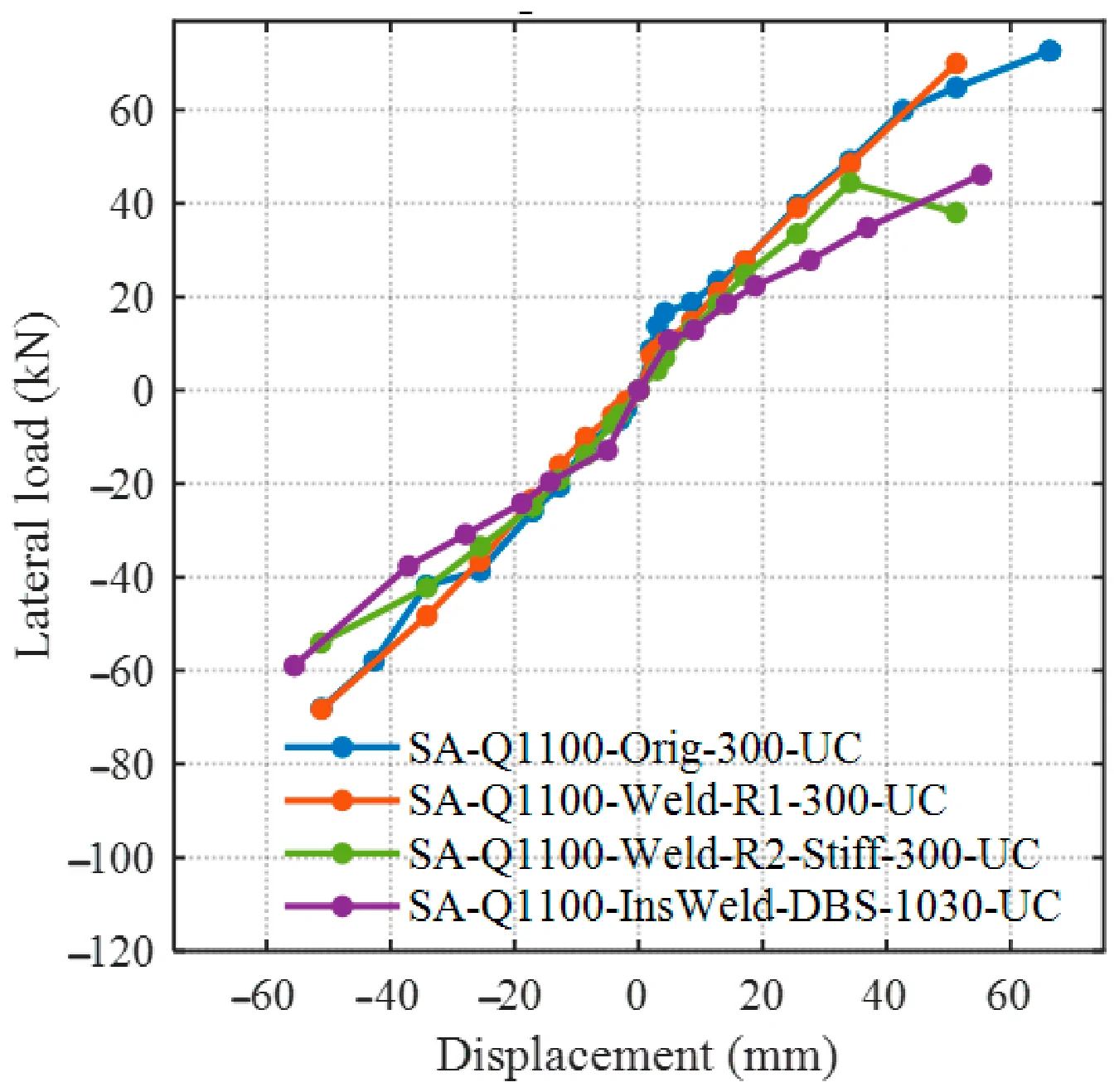
Backbone curves of SA-Q1100 box-section specimens under different welding repair and strengthening measures.

Figure 13 presents the backbone curves, which reveal systematic changes in the hysteretic behavior of the extended box-flange box-section specimen SA-Q1100 across its original state (SA-Q1100-Orig-300-UC), the initial weld repair (SA-Q1100-Weld R1-300-UC), the subsequent repair with additional stiffeners (SA-Q1100-Weld R2-Stiff-300-UC), and the final repair employing inserted welding alongside a double-bottom-plate (DBS) reinforcement (SA-Q1100-InsWeld-DBS-1030-UC). At a drift ratio of 2%, all repaired specimens showed a marked loss of lateral load capacity, and stiffness deteriorated more rapidly as the number of repair cycles increased, suggesting that cumulative damage in the WAZ caused by successive welding may adversely impact the plastic energy dissipation ability of the steel. The final drift ratios for the original, first-repair, second-repair (stiffener-reinforced), and third-repair (inserted welding + DBS) specimens were 4%, 5%, 6%, and 5%, respectively. Despite the repair history typically diminishing the overall stiffness of the component, the implementation of suitable reinforcement techniques, such as stiffeners and a dual bottom plate, may augment—its deformability within the tested conditions, resulting in an ultimate drift ratio increase of 25% to 50% compared to the original condition, with the second repair strategy demonstrating the most pronounced enhancement. In general, the implementation of either stiffener reinforcement or inserted welding, along with a double-bottom-plate enhancement approach, may enhance the ductility and fracture resistance of UHSS members subjected to intense seismic forces within the tested conditions, thus offering a feasible technical avenue for post-earthquake retrofitting design; however, further validation is required before general design recommendations can be made.

### 4.2. Influence of Weld Repair on Cyclic Response

**Figure 14:**
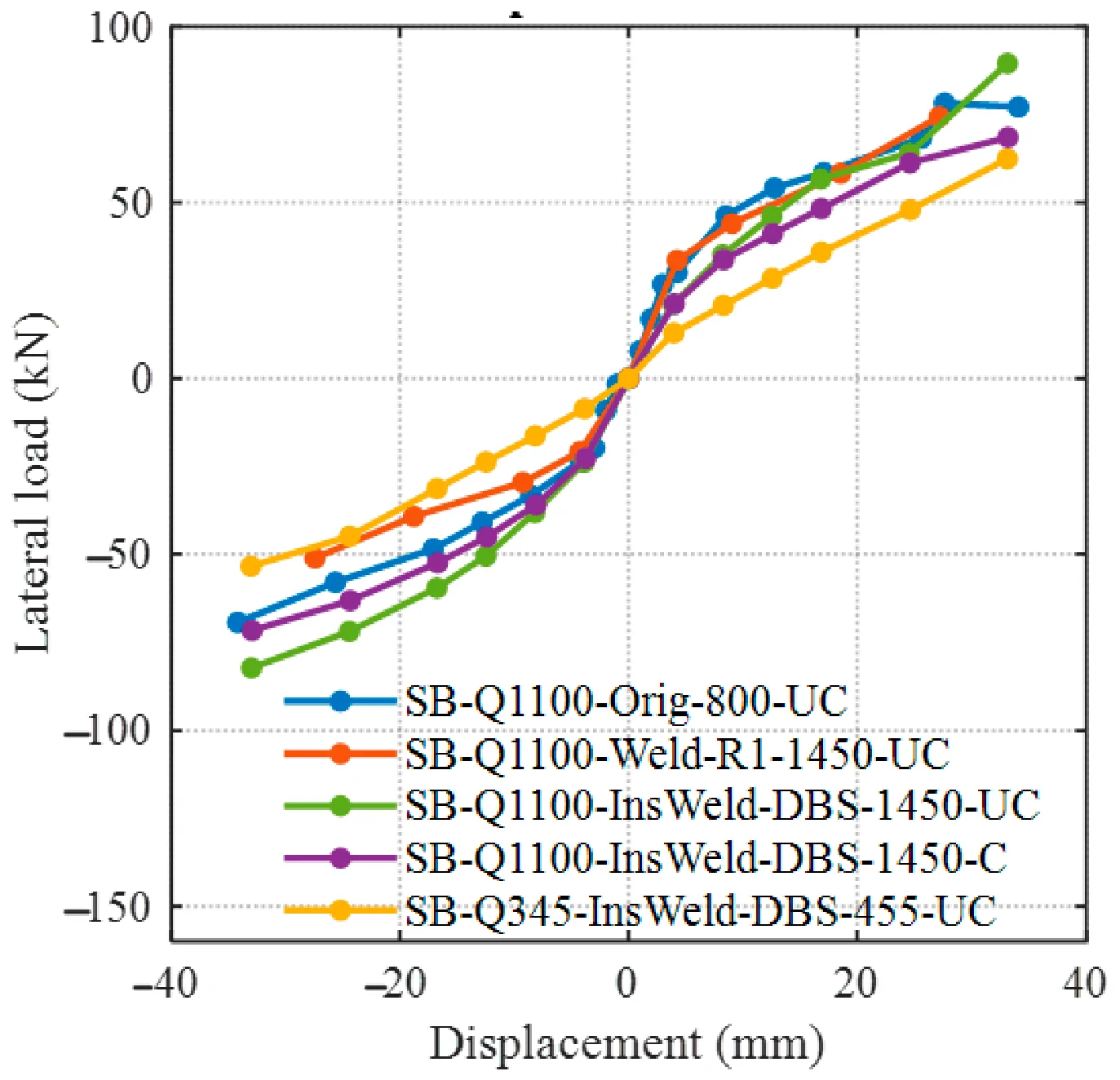
Backbone curves of SB-Q1100 and SB-Q345 specimens considering varying welding quality, top restraint, and steel grades.

Figure 14 illustrates the hysteretic behaviors of the SB Q1100 specimens subjected to various repair conditions: the unaltered specimen (SB-Q1100-Orig-800-UC), the specimen repaired with initial welding (SB-Q1100-Weld R1-1450-UC), and the specimen undergoing a second repair featuring inserted welding and a double bottom plate reinforcement (SB-Q1100-InsWeld-DBS-1450-UC). It should be noted that the axial load increased from 800 to 1450 kN after the first repair; therefore, the number of repair passes is not the only variable among these specimens. Under positive loading conditions, the lateral load capacity decreased slightly as the number of repair cycles increased at the same displacement amplitudes; nevertheless, the decrease was minimal. In the negative loading direction, the capacity significantly decreased following the initial repair, although rebounded after the subsequent repair (inserted welding + DBS), which is consistent with a direction-dependent response; however, other parameters also vary. These results suggest that the weld joint at the member base may be an important factor, although the observed differences should be interpreted within the tested parameter range.

The ultimate drift ratios for the original, first repair, second repair (without top lateral contact constraint), and second repair with top lateral contact constraint (SB-Q1100-InsWeld-DBS-1450-C) specimens were 2%, 5%, 7%, and 9%, respectively. These findings indicate that, within the tested specimens, base repair (welding + DBS) was associated with improved joint deformability. Furthermore, with the same vertical load, the specimen featuring top lateral contact constraint demonstrated an enhanced ultimate drift ratio from 7% to 9%, suggesting that, under the tested conditions, the incorporation of lateral support at the column apex was associated with enhanced overall seismic resilience. Because No. 8 is a retest of the repaired configuration rather than an independent companion, this comparison is not a replicate-based estimate of the restraint effect.

Moreover, in comparison with the SB Q1100 series, the SB-Q345-InsWeld-DBS-455-C specimen demonstrated the minimal lateral force and rigidity at an equivalent displacement level. However, this comparison also involves differences in axial load ratio and section geometry; therefore, it should not be interpreted as direct evidence that Q1100 offers superior seismic performance to Q345.

### 4.3. Effect of Sectional Dimensions on Skeletal Response of SA-Q1100, SB-Q1100 and SC-Q1100 Components

**Figure 15:**
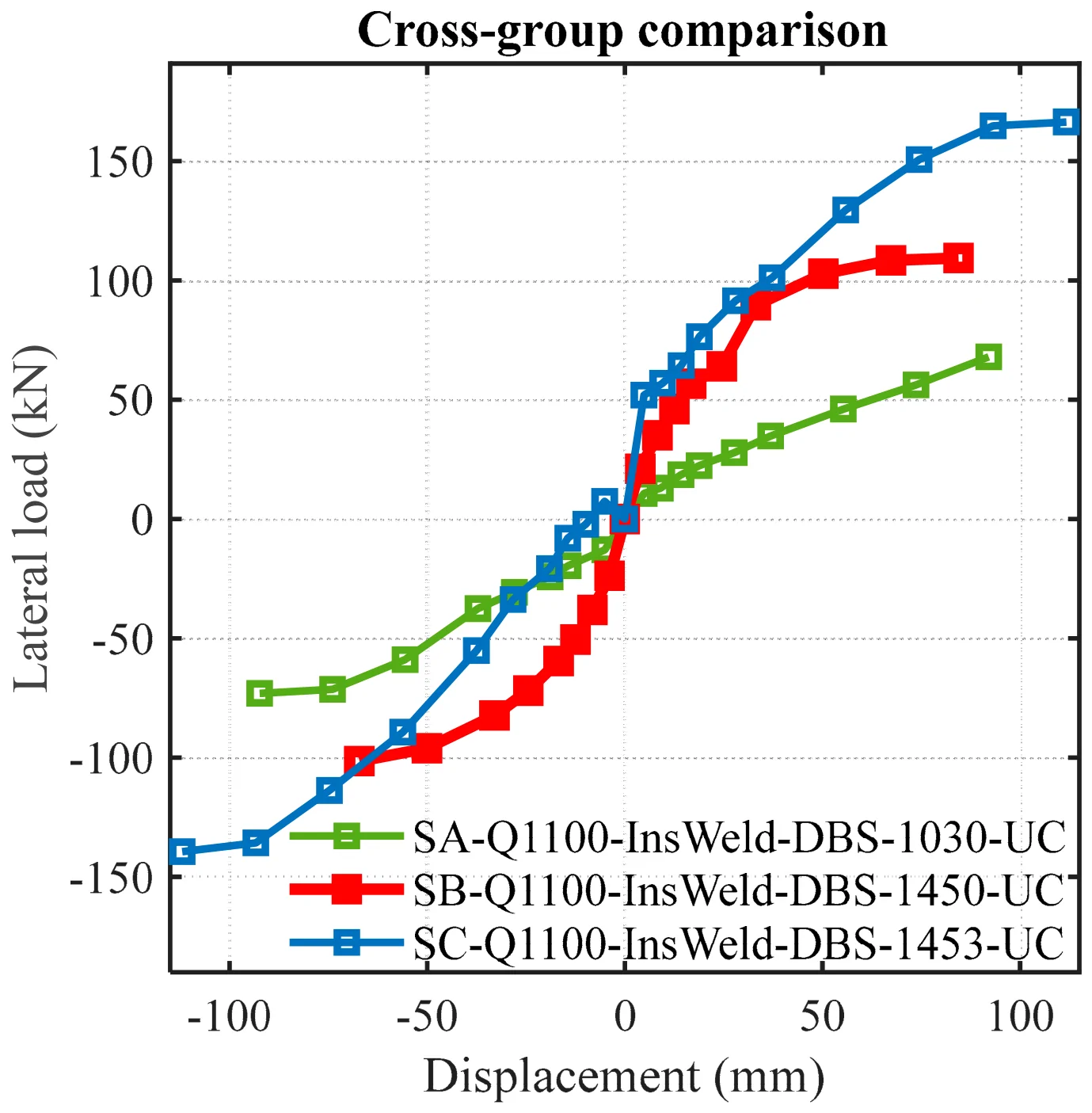
Comparison of backbone curves for SA, SB, and SC specimens with varied extended-flange configuration.

Figure 15 illustrates the backbone curves of three evaluated specimens: SA/SB/SC-Q1100-InsWeld-DBS-1450-UC. The ultimate drift ratios of these specimens were roughly 5%, 7%, and 6%, respectively, which may be partly associated with the absence of extended flanges in the SA member; however, these specimens also differ in axial load ratio and section dimensions, so the comparison should be interpreted with caution.

The lateral load resistance of the SA-Q1100 specimen remained below that of the SB-Q1100 and SC-Q1100 specimens under equivalent displacement amplitudes across the entire loading history. This trend is consistent with the incorporation of extended flanges, improving the load-bearing capability, although other parameters also vary among the specimens.

Both the SA-Q1100 and SB-Q1100 samples exhibited clearly delineated antisymmetric reactions throughout both positive and negative loading cycles. In comparison, the SC-Q1100 specimen, due to its asymmetrical extended-flange design, with a broader flange on the positive loading side and a narrower one on the negative loading side, demonstrated—a significantly greater capacity in the positive direction compared to its negative direction equivalent, leading to a distinctly non-symmetric backbone curve. This indicates that uneven flange width allocation contributes to the shape of the hysteretic envelope. Additionally, in comparison to the SB-Q1100 specimen, the SC-Q1100 specimen had solely a localized geometric alteration, specifically an extra 50 mm + 50 mm extended flange on one side. Notwithstanding this slight modification, the SC specimen exhibited enhanced positive lateral strength, but a lower ultimate drift ratio (6%) than the SB-Q1100 specimen (7%); its ultimate drift ratio was slightly higher than that of SA-Q1100 (5%). These observations are consistent with the influence of extended flange geometry on the hysteretic behavior, but they should be regarded as trends within the tested specimens rather than definitive causal proof.

### 4.4. Strength and Stability Evaluation with Existing Studies

#### 4.4.1. Strength Evaluation for Beam-Columns with Steel Box Sections with Extended Flanges

According to Clause 7.1.1 of JGJ/T 483 2020 [14], the conceptual foundation for the verification of section strength in beam-columns subjected to biaxial bending is formulated as follows.

In the examined box section specimens of this research, the strength criterion is articulated as a linear combination of axial and flexural stress resultants, as expressed in Equation (1) of [14]. As per the cross-section classification criteria for axially loaded compression elements (Table 5.2.2-1 of [14], plate thickness *t* < 40 mm), welded I-sections and welded box-sections are categorized as Class b concerning the y-axis. Regarding the x-axis, steel grades Q620, Q690, and higher are classified as Class a, whereas grades beneath Q620 are categorized as Class b. In the current computational framework, the Class b buckling curve is applied to both principal axes as a conservative simplification, which yields a consistent stability assessment throughout all ten design configurations.

For Q460 and Q460GJ steels, these parameters are established in accordance with GB 50017 [30], whereas for all other high-strength steel grades, including the Q1100 material utilized in this study, they are required to be set at 1.0, thus mandating a purely elastic section design. In the particular instance of the box-shaped cross sections analyzed herein, where the predominant bending force is exerted around the major axis (*M_y_* = 0), the overarching formula simplifies to

(2) $$S_{\mathrm{total}}=\frac{N}{A_{n}f}+\frac{M_{x}}{\gamma_{x}W_{\mathrm{nx}}f}\leq 1.0$$

where *N* represents the design axial force, and *M_x_* and *M_y_* denote the design bending moments around the major and minor primary axes, *A_n_* signifies the net cross-sectional area, and Wnx and Wny are the corresponding net section moduliand. The coefficients *γ_x_* and *γ_y_* are the plastic growth factors associated with the section moduli concerning the *x*- and *y*-axes, respectively. Equation (2) functions as the principal strength assessment in the ensuing parametric analysis. This provision ensures that local material yielding is preventede under high compressive stresses before global instability occurs, hence offering an additional lower bound protection for the design of the member.

Table 9 presents the computed strength interaction ratio *S*_total_ as per Equation (2), which indicates the local material yielding capability under simultaneous axial force and bending moment.

Only Scheme No. 9 (SB-Q345-InsWeld-DBS-455-C) surpasses the unity check, exhibiting a ratio of 1.004, which signifies a slight overstress regarding section strength. This is mainly attributable to the reduced yield strength (345 MPa) coupled with a comparatively elevated axial load (455 kN), where the plastic development factor *γ_x_* = 1.05 fails to offset the decreased material resistance.

The remaining nine schemes demonstrate strength ratios of *S*_total_ well below 1.0, with values spanning from 0.402 (No. 7) to 0.886 (No. 2). Significantly, Specimens No. 6 and No. 7 (SB series with a 1450 kN axial load) have the lowest strength ratios (~0.42–0.44), indicating that their design is dictated by instability limitations rather than yielding.

In instances involving high-strength steel (Q1100), the strength utilization remains moderate (≤0.886)remains despite substantial axial loads (e.g., No. 10: 1453 kN), indicating that the elevated yield stress offers significant protection against structural failure.

Among the ten tested schemes, only No. 9 exceeded the strength criterion; for the nine Q1100 schemes, strength was satisfied and stability was more critical.

#### 4.4.2. Stability Evaluation for Steel Box Sections Beam-Columns with Extended Flanges

(1) **In-Plane Stability**

The in-plane stability criterion [14], expressed as

(3) $$R_{\mathrm{in}\text{-}\mathrm{plane}}=\frac{N}{\varphi_{x}\mathrm{Af}}+\frac{\psi \beta_{\mathrm{mx}}M_{x}}{\gamma_{x}W_{1x}f(1-0.8N/{N^{\prime }}_{\mathrm{EX}})}\leq 1.0$$

*R*_in-plane_ concurrently considers the principal compressive stress, the secondary enhancement resulting from the interaction between axial force and bending moment, as well as the advantageous (or adverse) influences represented by the correction factor *ψ*.

The in-plane stability assessment in Table 10 excludes Nos. 4, 9, and 10 from the permissible range, exhibiting interaction ratios of 1.101, 1.093, and 1.054. Their exceedances do not possess a uniform governing mechanism. In No. 4, the elevated axial load ratio (*N*/(*φx Af*) = 0.601) predominates the compressive component, propelling the ratio over the threshold. Specimen No. 8 is influenced by a blend of diminished material strength (345 MPa), considerable slenderness (*λ_x_* = 30.75), and significant bending requirements, resulting in the highest interaction ratio of all samples. Number 10 is governed by moment control, as it experiences the greatest bending moment (238.1 kN·m); its increased section modulus (*W_c_* = 322,216 mm^3^) mitigates but does not completely eradicate the exceedance.

The remaining seven specimens (Nos. 1, 2, 3, 5, 6, 7, and 8) meet the criteria, exhibiting ratios ranging from 0.693 to 0.947. Nevertheless, Nos. 2 and 3 are near the threshold (0.947 and 0.918), providing minimal leeway for supplementary axial load or bending. The present checks therefore indicate that in-plane stability is influenced not solely by section capacity, but by the synergistic impacts of axial compression, material strength, slenderness, and bending requirements. Samples approaching the threshold must be handled with care, as even slight variations in load could render them non-compliant.

(2) **Out-of-Plane Stability**

The out-of-plane stability validation [14] is governed by

(4) $$R_{\mathrm{out}\text{-}\mathrm{plane}}=\frac{N}{\varphi_{y}\mathrm{Af}}+\eta \frac{M_{x}}{\varphi_{b}\gamma_{x}W_{1x}f}\leq 1.0$$

where *φ_y_* and *φ_b_* are the stability coefficients, *η*(0.7) is the interaction coefficient, and *γ_x_* is the moment amplification factor.

Table 10 presented the findings of the out-of-plane stability validation based on Equation (4). The out-of-plane assessment reveals that only No. 8 and No. 9 are non-compliant, with ratios of 1.027 and 1.032. In No. 8, the bending-moment value (0.819) significantly exceeds the axial value (0.208), suggesting that the lateral-torsional resistance is inadequate for the stiffening configuration implemented in this design. No. 9 does not meet the in-plane or out-of-plane standards; its inferior material quality and comparatively limited lateral flexural rigidity (*φ_y_* = 0.450) are the primary factors. Numbers 6 and 7 are still deemed technically satisfactory (0.978 and 0.949), although their closeness to one provides minimal margin, meaning that even a slight rise in bending demand or a decrease in lateral support could instigate out-of-plane buckling. The other six schemes (Nos. 1–5 and 10) fall well short of the threshold, with ratios ranging from 0.521 to 0.777. The findings indicate that the stability out of the plane is predominantly influenced by the bending-moment factor instead of axial compression, and that sufficient lateral support is crucial for repaired components, particularly those exhibiting low strength or restricted lateral stiffness.

(3) **Overall Stability Assessment**

The integrated in-plane and out-of-plane evaluations indicate that Nos. 1, 2, 3, 5, 6, and 7 meet both standards; however, Nos. 6 and 7 are precariously close to failing out-of-plane, leaving almost no margin. Numbers 4, 8, 9, and 10 do not meet the compliance criteria. Among the ten tested schemes, No. 9 is the sole instance that concurrently exhibits deficiencies in strength, in-plane stability, and out-of-plane stability, indicating a code-based deficiency for the tested configuration. The findings indicate for the tested schemes that the stability criteria outlined in JGJ/T 483-2020 [14] are essential; solely depending on section strength would erroneously validate Nos. 4, 8, and 10, which are classified as unstable by the code checks; this does not by itself establish the observed failure mechanism.

The design implications are twofold. In-plane stability is primarily influenced by the axial load ratio and bending moment, with second-order effects becoming significant for severely loaded short elements. Out-of-plane stability is primarily influenced by lateral flexural rigidity and lateral-torsional strength; closed box sections gain an advantage at η = 0.7; this benefit may, however, be reduced by large bending moments;,. In instances of non-compliance, adherence can be attained by augmenting the section modulus (specifically *W*_1x_ and *W*_2x_) to mitigate moment-induced stresses, decreasing the effective buckling length or incorporating intermediate lateral supports, or enhancing the material grade (for example, from Q345 to Q550 or superior for No. 9) to improve strength and stability. No uncertainty analysis was performed for the near-unity results; the values 1.004, 1.027, and 1.032 should therefore be treated as marginal rather than definitive.

#### 4.4.3. Comparative Assessment and Governing Factors

Table 11 identifies the governing design basis for each scheme. Nos. 1–3 are controlled by the in-plane interaction, whereas No. 5 is controlled by the out-of-plane interaction; their computed ratios (*R*_in-plane_ = 0.69–0.95 and *R*_out-plane_ = 0.52–0.78) generally lie below unity by margins comparable to or larger than the estimated ±5–8% input-sensitivity band, so the ranking indicates the relatively more critical direction rather than a definitive pass verdict. No. 4 (*R*_in-plane_ = 1.101) and No. 10 (*R*_in-plane_ = 1.054) exceed unity by about 10% and 5%, respectively, and are therefore reported as marginal, with the high axial load ratio and large in-plane bending moment identified as the dominant contributors, respectively. Nos. 6–8 are governed by out-of-plane action, with *R*_out-plane_ = 0.978, 0.949 and 1.027; these values lie within or very close to the uncertainty band and are treated as marginal rather than definitive failures. No. 9 gives *R*_in-plane_ = 1.093 and *R*_out-plane_ = 1.032, the smallest margin in the series, which warrants attention but does not support a definitive inadequacy verdict.

For Nos. 7 and 8, the measured response and the code-based indices are reported separately. The two tests share the same member and repaired configuration under 1450 kN vertical load and differ only in the top lateral boundary condition (free in No. 7 and contact-constrained in No. 8). No. 8 is therefore a retest of the same configuration rather than an independent replicate. The code-based indices depend on the effective length adopted for each boundary condition: the slenderness ratio is 100.98 for No. 7 and 30.75 for No. 8, yielding interaction ratios of 0.422 and 0.545, respectively. These ratios are normalized design demands, not measured resisted moments; the large difference between the two tests therefore reflects the change in assumed buckling length induced by the lateral restraint, rather than a change in material or cross-sectional capacity. Accordingly, comparisons between Nos. 7 and 8 are based on the observed response, while the code-based indices are used only to verify the applicable design provisions.

## 5. Conclusions

This study experimentally investigated the cyclic behavior and observed failure modes of Q1100 ultra-high-strength steel box-section beam-columns with extended flanges subjected to multiple weld repair histories. Ten test schemes were conducted on four physical members; the schemes sharing a member are sequential and are not independent replicates. The following conclusions are drawn:

(1) All tested Q1100 schemes failed by fracture initiating at the lower plate/weld region, even with stiffeners, double bottom plates, or inserted end welding. The tested Q345 specimen showed local buckling followed by weld tearing. The observed fracture-controlled response may be associated with limited member-level deformation capacity.
(2) The observed degradation in hysteretic response is consistent with cumulative WAZ effects induced by repeated welding; however, the repair sequences were confounded with changes in axial load, strengthening, and boundary condition. After the second repair, hysteresis loops became asymmetric, with positive/negative load and energy dissipation decoupled. Strengthening partly offset this within the tested specimens: stiffeners raised ultimate drift from 4% to 6%; inserted end welding plus double bottom plates restored strength and deformation; top lateral constraint was associated with an increase in SB-Q1100 drift from 7% to 9% and with increased cumulative energy dissipation, partly because more cycles were sustained. Because No. 8 is a retest of the No. 7 repaired configuration, this comparison is not a replicate-based estimate.
(3) In the tested specimens, extended flanges were associated with increased lateral capacity. Unequal flange widths in SC were associated with initial load offset and asymmetric envelopes. SA, without extended flanges, had the lowest resistance and deformation. At similar axial load ratios, the Q345 specimen had fuller loops and better plastic dissipation, while the Q1100 specimen carried about three times the absolute load; however, axial load ratio and section dimensions also differ, so this is not a direct material-grade comparison. A strength–ductility trade-off was observed within the test matrix.
(4) JGJ/T 483-2020 checks showed section strength alone would accept all Q1100 specimens, but several failed in-plane or out-of-plane stability; the Q345 specimen failed strength and both stability checks. Among the ten tested schemes, stability checks governed several repaired Q1100 specimens; generalization beyond this test matrix requires further validation. Compliance for the tested noncompliant cases requires larger section modulus, reduced effective length, or higher steel grade. The near-unity values should be treated as marginal because no uncertainty analysis was performed.

Overall, within the tested conditions, satisfactory performance was observed in specimens with effective strengthening and lateral restraint. Because WAZ damage was not directly characterized, the physical members were reused, and no repeated tests under identical conditions were performed, these conclusions are limited to the present test matrix; further microstructural, fractographic, welding-process, NDT, boundary-calibration, and repeat-test validation is required.

## Figures and Tables

**Table 1:** Parameters of Q1100 and Q345 specimens.

Indicator	*E*/GPa	*σ*_0.2_/MPa	*σ*ᵤ/MPa	*ε*ᶠ/%	*n*ᶠ
Q1100	206	1210	1470	11	0.06
Q345	206	345	470	12	/

**Table 2:** Nominal section dimensions of the tested specimens.

Specimen	*B* (mm)	*H* (mm)	*t_f_* (mm)	*b_s_*_0_ (mm)	*t_s_*_0_ (mm)	*b_ef_*_1_ (mm)	*b_ef_*_2_ (mm)	*B*_1_ (mm)	*B*_2_ (mm)	*A* (mm^2^)
SA	112	160	8	22	6	0	0	112	112	4360
SB	112	160	8	22	6	55	55	222	222	6120
SC	112	160	8	22	6	105	55	322	222	6920

**Table 3:** Specimen matrix.

No.	Label	Physical Member	Test Date	Description
1	SA-Q1100-Orig-300-UC	SA-Q1100	28 December 2024	SA series, original, vertical 300 kN, top lateral free (no lateral restraint)
2	SA-Q1100-Weld-R1-300-UC	SA-Q1100	30 December 2024	SA series, 1st welding repair after bottom fracture, vertical 300 kN, top lateral free; same physical member as No. 1
3	SA-Q1100-Weld-R2-Stiff-300-UC	SA-Q1100	7 January 2025	SA series, 2nd welding repair + end stiffeners, vertical 300 kN, top lateral free; same physical member as No. 1
4	SA-Q1100-InsWeld-DBS-1030-UC	SA-Q1100	7 July 2025	SA series (Q1100), inserted end welding + DBS, vertical 1030 kN, top lateral free; same physical member as No. 1
5	SB-Q1100-Orig-800-UC	SB-Q1100	3 April 2025	SB series, original, vertical 800 kN, top lateral free
6	SB-Q1100-Weld-R1-1450-UC	SB-Q1100	8 May 2025	SB series, 1st welding repair with high-strength wire, vertical 1450 kN, top lateral free; same physical member as No. 5
7	SB-Q1100-InsWeld-DBS-1450-UC	SB-Q1100	28 May 2025	SB series (Q1100), inserted end welding + DBS, vertical 1450 kN, top lateral free (unconfined); same physical member as No. 5
8	SB-Q1100-InsWeld-DBS-1450-C	SB-Q1100	30 May 2025	SB series (Q1100), same repaired configuration as No. 7 but top lateral contact constrained, vertical 1450 kN; retest, not an independent replicate
9	SB-Q345-InsWeld-DBS-455-C	SB-Q345	30 May 2025	SB series (Q345), inserted end welding + DBS, top lateral contact constrained, vertical 455 kN; separate Q345 member
10	SC-Q1100-InsWeld-DBS-1453-UC	SC-Q1100	7 July 2025	SC series (Q1100), inserted end welding + DBS, vertical 1453 kN, top lateral free; separate SC member

*Note: DBS = double bottom plates + stiffeners; UC = top lateral free; C = top lateral contact constrained.*

**Table 4:** **Parameters of the two pseudo-static testing systems** and their test scheme assignments.

System	Transverse Actuator	Vertical Actuator	Applied Schemes
System 1	500 kN (static & dynamic) Stroke: 254 mm Max. pressure: 20.7 MPa	1000 kN (max.) Stroke: 200 mm Class 1 accuracy	No. 1–3, No. 5 (UC)
System 2	Static 1290 kN/dynamic 870 kN Max. pressure: 21 MPa	Static 2100 kN/dynamic 1600 kN Stroke: ±250 mm Max. pressure: 21 MPa	No. 4, No. 6–10 (UC/C)

**Table 5:** **Descriptive statistics of vertical loading** for the selected schemes (each system compared with its own target; no cross-system equivalence is implied) (*p*_Normality_: 0.001, Method: Box–Cox).

Loading System	Scheme Example	Target/kN	Samples	Mean/kN	*Std*_Overall_/kN
System 1	SA-Q1100-Orig-300-UC	300	51,678	297.957	10.821
System 1	SA-Q1100-Weld R1-300-UC	300	57,075	299.380	12.002
System 2	SB-Q1100-Weld R1-1450-UC	1450	234,400	1450.505	6.935
System 2	SC-Q1100-InsWeld DBS-1453-UC	1453	36,125	1451.475	1.520

**Table 6:** Summary of specimen properties and load parameters.

No.	*λ*	*N_v_* (kN)	*μ*
1	82.45	300	0.057
2	82.45	300	0.057
3	82.45	300	0.057
4	88.96	1030	0.195
5	93.59	800	0.108
6	100.98	1450	0.196
7	100.98	1450	0.196
8	30.75	1450	0.196
9	30.75	455	0.216
10	96.34	1453	0.174

**Table 7:** Comparison of ultimate drift ratios for ten test schemes.

No.	1	2	3	4	5	6	7	8	9	10
*DR*/%	4%	5%	6%	5%	2%	5%	7%	9%	8%	6%

**Table 8:** Cyclic test results: cycles to failure and cumulative dissipated energy for all specimens.

No.	Cycles	Total Energy (kN·mm)
1	22	13,939
2	25	17,157
3	22	43,292
4	20	35,639
5	9	11,091
6	13	39,990
7	18	48,815
8	25	227,786
9	29	285,545
10	23	88,686

*Note: One cycle is defined as one complete positive loading–unloading excursion followed by one complete negative loading–unloading excursion for the integration.*

**Table 9:** Computed strength verification indices for ten schemes.

No	1	2	3	4	5	6	7	8	9	10
*S*_total_	0.693	0.886	0.855	0.719	0.584	0.442	0.402	0.513	1.004	0.863

**Table 10:** Computed code-based interaction checks for the ten schemes.

No	Stability Validation [14]		*R*_Interaction_
	*R*_in-plane_	*R*_out-plane_	
1	0.766	0.521	0.712
2	0.947	0.656	0.911
3	0.918	0.635	0.880
4	1.101	0.628	0.778
5	0.72	0.777	0.610
6	0.733	0.978	0.467
7	0.693	0.949	0.422
8	0.803	1.027	0.545
9	1.093	1.032	1.070
10	1.054	0.684	0.925

**Table 11:** Governing code-based interaction check and separately observed failure mode for the ten schemes.

Scheme	Governing Code-Based Interaction (Design Check)	Remarks and Experimentally Observed Failure Mode
1–3	In-plane (No. 1–3)	In-plane code check governs (*R*_in-plane_ =0.766, 0.947, 0.918 > *R*_out-plane_ =0.521, 0.656, 0.635). Fracture at the lower plate/weld region.
4	In-plane stability	*R*_in-plane_ = 1.101 exceeds unity by about 10%, which is of the same order as the plausible input-sensitivity band; the case is therefore reported as marginal rather than as a definitive failure, and the high axial load ratio (*μ* = 0.195) is identified as the dominant contributor.
5	Out-of-plane (No. 5)	Out-of-plane code check governs (*R*_out-plane_ = 0.777 > *R*_in-plane_ = 0.720). Fracture at the lower plate/weld region.
6–7	Out-of-plane (marginal)	*R*_out-plane_ = 0.978 (No. 6) and 0.949 (No. 7) lie within roughly 2–5% of unity, i.e., inside the plausible input-sensitivity range; the response is described as marginal (lateral buckling close to the limit) and no definitive pass is claimed.
8	Out-of-plane stability	*R*_out-plane_ = 1.027 exceeds unity by less than 3%, which is well inside the uncertainty band and therefore not distinguishable from unity; the case is treated as marginal, with moment amplification in the lateral direction identified as the governing mechanism.
9	Strength & both stabilities	*R*_in-plane_ = 1.093 and *R*_out-plane_ = 1.032 exceed unity by 3–9%, i.e., of the order of the input-sensitivity band; the margin is the smallest in the series and warrants attention (material upgrade or section enlargement may be considered), but a definitive inadequacy verdict is not supported by the present data.
10	In-plane stability	*R*_in-plane_ = 1.054 exceeds unity by about 5%, comparable to the plausible input-sensitivity band; the case is reported as marginal, with the large in-plane bending moment identified as the dominant action.

## Data Availability

The original contributions presented in this study are included in the article. Further inquiries can be directed to the corresponding author.
